# Association mapping of loci controlling genetic and environmental interaction of soybean flowering time under various photo-thermal conditions

**DOI:** 10.1186/s12864-017-3778-3

**Published:** 2017-05-26

**Authors:** Tingting Mao, Jinyu Li, Zixiang Wen, Tingting Wu, Cunxiang Wu, Shi Sun, Bingjun Jiang, Wensheng Hou, Wenbin Li, Qijian Song, Dechun Wang, Tianfu Han

**Affiliations:** 1 College of Agriculture, Northeast Agricultural University, Harbin, 150030 Heilongjiang China; 2 MOA Key Laboratory of Soybean Biology (Beijing), Institute of Crop Science, the Chinese Academy of Agricultural Sciences, 12 Zhongguancun South Street, Beijing, 100081 China; 3 Department of Plant, Soil and Microbial Sciences, Michigan State University, 1066 Bogue St., Rm. A384-E, East Lansing, MI 48824-1325 USA; 4 Soybean Genomics and Improvement Laboratory, US Department of Agriculture, Agricultural Research Service (USDA-ARS), 10300 Baltimore Ave, Beltsville, MD 20705 USA

**Keywords:** Soybean (*Glycine max*), Genetic architecture, Gene by environment interaction, Flowering time, Photo-thermal condition

## Abstract

**Background:**

Soybean (*Glycine max* (L.) Merr.) is a short day plant. Its flowering and maturity time are controlled by genetic and environmental factors, as well the interaction between the two factors. Previous studies have shown that both genetic and environmental factors, mainly photoperiod and temperature, control flowering time of soybean. Additionally, these studies have reported gene × gene and gene × environment interactions on flowering time. However, the effects of quantitative trait loci (QTL) in response to photoperiod and temperature have not been well evaluated. The objectives of the current study were to identify the effects of loci associated with flowering time under different photo-thermal conditions and to understand the effects of interaction between loci and environment on soybean flowering.

**Methods:**

Different photoperiod and temperature combinations were obtained by adjusting sowing dates (spring sowing and summer sowing) or day-length (12 h, 16 h). Association mapping was performed on 91 soybean cultivars from different maturity groups (MG000-VIII) using 172 SSR markers and 5107 SNPs from the Illumina SoySNP6K iSelectBeadChip. The effects of the interaction between QTL and environments on flowering time were also analysed using the QTXNetwork.

**Results:**

Large-effect loci were detected on Gm 11, Gm 16 and Gm 20 as in previous reports. Most loci associated with flowering time are sensitive to photo-thermal conditions. Number of loci associated with flowering time was more under the long day (LD) than under the short day (SD) condition. The variation of flowering time among the soybean cultivars mostly resulted from the epistasis × environment and additive × environment interactions. Among the three candidate loci, i.e. Gm04_4497001 (near *GmCOL3a*), Gm16_30766209 (near *GmFT2a* and *GmFT2b*) and Gm19_47514601 (*E3* or *GmPhyA3*), the Gm04_4497001 may be the key locus interacting with other loci for controlling soybean flowering time.

**Conclusion:**

The effects of loci associated with the flowering time of soybean were dependent upon the photo-thermal conditions. This study facilitates the understanding of the genetic mechanism of soybean flowering and molecular breeding for the improvement of soybean adaptability to specific and/or broad regions.

**Electronic supplementary material:**

The online version of this article (doi:10.1186/s12864-017-3778-3) contains supplementary material, which is available to authorized users.

## Background

As a short-day and temperate plant, soybean (*Glycine max*(L.) Merr.) is sensitive to photo-thermal conditions during flower initiation and development [1–3]. The responses of soybean cultivars to photo-thermal conditions determine the zone of their adaptation and affect yield, plant height, seed quality, etc. [4, 5].

Flowering time is one of the most important traits associated with seed yield and adaptation of soybean. Soybean flowering time is regulated by both genetic and environmental factors [6, 7]. At least 11 major loci control flowering time and maturity in soybean, including *E1*– *E10* [8–17] and *J* [18]. Among them, six genes (*E1*, *E2*, *E3*, *E4 E9* and *J*) have been cloned or identified. *E1* was reported to be a legume-specific transcription factor which could delay soybean flowering time in long-day conditions [19]. *E2* was identified to be an ortholog of the *Arabidopsis GIGANTEA* gene [20]. *E3* and *E4* were confirmed to be homologs of *PHYA* [21]. *E9* was recently identified as *GmFT2a*, an ortholog of *Arabidopsis FT* [22]. *J* was the dominant functional allele of *GmELF3* [23]. *GmFT5a* was also identified as a key gene to regulate soybean flowering time [24]. Other orthologs of *Arabidopsis* flowering genes such as *GmCOLs* [25], *GmSOC1* [26], and *GmCRY* [27], and many other genes controlling flowering time have also been identified [28].

Environmental factors, especially photoperiod and temperature, play important roles in flowering time. In previous studies, short day and high temperature accelerated the process from emergence to first flowering of soybean, whereas long day and low temperature delayed flowering time [2, 3, 7]. The interaction between photoperiod and temperature also influences soybean flowering time [2, 3, 7]. However, the genetic mechanism of photo-thermal effects on soybean flowering time is not well documented.

The interaction between gene and environment underlying flowering time has been well elucidated in *Arabidopsis thaliana* [29], *Boechera stricta* [30] and other species. In soybean, the effects of the genes on flowering time and maturity are influenced by environmental conditions [1]. Previous analysis of 39 near-isogenic lines (NILs) with 6 *E* genes (*E1*, *E2*, *E3*, *E4*, *E5* and *E7*) indicated that the effects of dominant alleles on flowering were enhanced in the long day and weakened in the short day [31]. The effects of *E* genes on maturity were also influenced by sowing seasons with different photo-thermal combinations. Each dominant gene had a smaller effect on maturity of soybean planted in summer than in spring [32]. The effects of the QTLs varied with the photoperiodic conditions [33] and latitudinal environments [34] and were population-specific, which enabled the plants to adjust to different climatic conditions [33, 34]. However, the responses of flowering time to photoperiod and temperature has not been systematically analysed.

QTXNetwork is a GPU parallel computing software to reveal genetic and environmental interaction underlying the genetic architecture of complex traits [35], the algorithm of the software was based on a mixed linear model. The software was used to study the genetic variations of lint yield and its component traits in cotton [35], and the chromium content and total sugar level in tobacco leaf [36].

The objectives of this study were to determine the variation of QTL effects under different photo-thermal environments and the interaction between the QTL and environments on soybean flowering time using a diverse set of soybean genotypes from different ecological regions.

## Methods

### Plant materials

The diversity panel used in this study consisted of 91 cultivars originating from different ecological regions in China (75 cultivars) and different maturity groups in the US (16 cultivars). The Chinese cultivars included six sowing season ecotypes, i.e., Northern Spring Sowing type (Nsp) (29 cultivars), Huang-Huai-Hai Spring Sowing type (Hsp) (4 cultivars), Huang-Huai-Hai Summer Sowing type (Hsu) (13 cultivars), Southern Spring Sowing type (Ssp) (13 cultivars), Southern Summer Sowing type (Ssu) (8 cultivars) and Southern Autumn Sowing type (Sau) (8 cultivars) covering a range of latitudes from 20°03’N to 50°15’N. The US cultivars were from different maturity groups (MG 0-VI) (Additional file 1: Table S1).

### Experimental design and phenotypic data collection

The pot experiments were conducted outdoor at the Institute of Crop Science, CAAS, Beijing, China (39°54’N, 116°46’E) during 2009 and 2010. In 2009, only 25 cultivars from different ecological regions were used (Additional file 1: Table S1). The pots were arranged in a completely randomized design with three replications in six photo-thermal environments. These cultivars were planted on May 4 (spring) and June 18 (summer) in 2009, and on April 10 (spring) and June 29 (summer) in 2010, so the plants could be exposed to low temperature (LT) by growing in the spring and high temperature (HT) in the summer [37]. Each replicate consisted of five seedlings with uniform growth in each pot. After the cotyledons were fully expanded (VC), the plants were placed in four different photoperiod treatments: short day (SD) (12 h), long day (LD) (16 h), natural day-length of spring sowing in Beijing (SP) and natural day-length of summer sowing in Beijing (SU). Under the SD treatment, seedlings were placed in the natural sunshine for 12 h, followed by 12 h in the darkness from 7 pm to 7 am. A platform truck was used to transfer the plants to the dark room. Under the LD treatment, plants were provided artificial light from 4 am to 6 am and from 6 pm to 8 pm. Incandescent bulbs with photosynthetically active radiation (PAR) at approximately 50 μmols^−1^m^−2^ placed above the canopy when the bulbs were the only source of light [37, 38]. The mean natural day-length of planting season (May 4- October 9) in Beijing was 13.82 h, and the longest (June 23) and shortest (October 9) day-length were 15.02 h and 11.45 h, respectively.

The field experiments were also conducted at the Institute of Crop Science, CAAS, Beijing, China in 2014 and 2015. These cultivars were planted on April 30 (spring) (14SP) and June 25 (summer) (14SU) in 2014, and on May 4 (spring) (15SP) and July 1 (summer) (15SU) in 2015. All lines were arranged in a completely randomized design with three replications.

During the experiment, the phonological stages of emergence (VE) and the beginning bloom (R1) were recorded as described by Fehr and Carviness (1977) [39] as well as Wu et al. (2015) [37].

### DNA extraction and genotyping

Genomic DNA was isolated from fresh leaves of five plants of each cultivar using the SDS (sodium dodecyl sulfate) method [40]. One hundred and seventy-two SSR makers associated with QTLs controlling phenological traits and other agronomic traits were selected according to previous studies (SoyBase (http://www.soybase.org)). SSR primers were from SoyBase (http://www.soybase.org). The PCR reaction mixture contained 100 ng of genomic DNA, 2 μl of 10 × PCR Buffer (+Mg^2+^), 2 μl of dNTPs (2 mM), 0.5 μl of SSR primer (10 mM), 0.2 μl of Taq polymerase (10 units/μl) and 13.8 μl of ddH_2_O in a total volume of 20 μl. The amplification program consisted of 94 °C for 5 min, 35 cycles of 94 °C for 30 s, 49 °C for 30 s, 72 °C for 45 s and 72 °C for 5 min. Then, the PCR products were separated on 6% w/v denaturing polyacrylamide gels, and the fragments were visualized by silver staining. The cultivars were also genotyped with Illumina BARCSoySNP6K iSelectBeadChip (Illumina, San Diego, Calif. USA) containing 5,403 SNPs selected from SoySNP50K [41]. After elimination of SNPs with missing allele >24%, or minor allele frequency <0.05 [42], a total of 5,107 SNPs remained (Additional file 2: Table S2). SSR and SNP data were used for association mapping, and the SNP data was used for QTXNetwork analysis.

### Genetic diversity and population structure analysis

The population structure was inferred from 63 SSR markers, which were randomly chosen and evenly distributed on 20 chromosomes (Additional file 2: Table S2), using the Bayesian Markov Chain Monte Carlo model via STRUCTURE v.2.3.1 software [43]. The K value (number of subpopulations) was set from 1 to 10 using a burn-in of 50,000, a run length of 100,000, and each K value was obtained with seven independent runs. The *ad hoc* quantity (*ΔK*) was estimated through the website (http://taylor0.biology.ucla.edu/structureHarvester) to determine the true K value [44]. The Q matrix was obtained by the CLUMPP software and by integrating the cluster membership coefficient matrices of replicated runs from STRUCTURE. A similar procedure described above was used for population structure analysis based on 5,107 SNP makers. A principal component analysis (PCA) for population structure was conducted by GenAlex 6.5 and the neighbour-joining tree was constructed by POWERMARKER v. 3.25 and MEGA 5. The genetic diversity of the panel was also analysed by POWERMARKER v. 3.25.

### The linkage disequilibrium and association analysis

The TASSLE v. 3.0 software was used to calculate the linkage disequilibrium (*r* ^2^) for all pairwise loci of the SNP markers [45]. The General Linear Model (GLM) and the Q matrix from STRUCTURE software were used to identify the association of 172 SSR and 5,107 SNP markers with flower time [46]. The Bonferroni-corrected thresholds for the *p*-value were used to determine the significance of association and were 2.90 × 10^−4^ (0.05/172), and 9.79 × 10^−6^ (0.05/5107) for SSR and SNP markers, respectively. Functional annotations of SNPs and SSRs were performed using the Phytozome database (https://phytozome.jgi.doe.gov) and SoyBase database (SoyBase (http://soybase.org).

### Association mapping based on the QTXNetwork

The QTXNetwork software was used to dissect the genetic architecture of the flowering time with 5,107 SNPs. Association mapping was performed using the mixed linear model with environment (E) as a fixed effect, and the loci effects (a, additive effect; aa, epistasis effect) and loci by environment interaction (ae, additive by environment interaction; aae, epistasis by environment interaction) as random effects [35]. The loci with –log_10_(*P*-value) > 3.0 in different environments were identified.

## Results

### The effects of photoperiod and temperature on flowering time in soybean

A wide range of phenotypic variation was observed in flowering time in the association panel across different photo-thermal conditions (Table 1). All cultivars can flower under the SD or natural-day condition regardless of the sowing season. However, some cultivars in the LD condition failed to flower at the harvest season. The soybean flowering time followed a normal distribution except for flowering time in natural day-length conditions, which was slightly skewed to the early flowering (Table 1, Additional file 3: Figure S1). The duration from emergence (VE) to the beginning bloom (R1) was shorter in the SD than that in the LD condition given the same sowing season. However, the time from emergence (VE) to the beginning bloom (R1) was accelerated in the HT compared with that in the LT under the same day-length.

**Table 1:** Descriptive statistics of soybean flowering time in different photo-thermal treatments

Year	Environment^a^	Min.	Max.	Mean ± SE	CV(%)	Skewness	Kurtosis
		(d)	(d)	(d)			
2009^b^	SD + LT	22.4	35.6	28.4 ± 0.7	11.85	0.47	0.16
	SD + HT	21.5	31.3	25.9 ± 0.6	10.60	0.17	−0.89
	LD + LT	30.0	>114.8^c^	>70.9 ± 5.0^c^	33.87	−0.10	−0.85
	LD + HT	24.0	>80.2^c^	>47.5 ± 3.6^c^	35.57	0.62	−0.47
	SP	28.5	133.6	52.4 ± 4.9	45.68	1.77	4.59
	SU	25.2	64.8	37.9 ± 2.8	35.11	−0.65	1.94
2010	SD + LT	24.0	35.1	29.0 ± 0.3	8.69	0.10	−0.27
	SD + HT	22.1	31.8	26.6 ± 0.2	7.74	0.18	−0.47
	LD + LT	26.7	>165.7^c^	>98.0 ± 3.9^c^	35.67	−0.03	−0.61
	LD + HT	26.6	>103.4^c^	>61.5 ± 2.3^c^	31.55	0.41	−0.28
	SP	25.3	137.5	53.9 ± 2.9	49.26	1.34	1.37
	SU	23.4	81.5	38.8 ± 1.2	29.18	1.15	1.56
2014	14SP	19.5	132.9	50.9 ± 3.2	58.39	1.12	0.25
	14SU	18.6	84.5	39.2 ± 1.6	39.51	0.95	0.25
2015	15SP	19.7	124.8	47.6 ± 2.9	57.91	0.99	−0.13
	15SU	19.1	76.0	36.3 ± 1.4	36.79	0.83	0.08

^a^SD, 12 h; LD, 16 h; LT, low temperature (spring sowing); HT, high temperature (summer sowing); SP, Spring sowing season with natural day-length in pot experiment; SU, Summer sowing season with natural day-length in pot experiment; 14SP, Spring sowing in 2014 field experiment; 14SU, Summer sowing season in 2014 field experiment; 15SP, Spring sowing season in 2015 field experiment; 15SU, Summer sowing season in 2015 field experiment

^b^A total of 91 cultivars were tested in the experiment in 2010, 2014 and 2015, and a subset of 25 cultivars from different maturity groups were used in the experiments in 2009, the cultivars were listed in the Additional file 1

^c^Some late cultivars failed to flower before the end of experiment. The flowering time of the latest-flowered cultivar in the same treatment was used as that of the un-flowered cultivars when calculating the means

Collectively, high temperature and short day had additive effects on accelerating the flowering time. The mean pre-flowering phase was the shortest in the SD + HT condition (25.9 d and 26.6 d in 2009 and 2010, respectively) and the longest in the LD + LT condition (70.9 d and 98.0 d or more in 2009 and 2010, respectively). These results suggest that flowering time can be greatly affected by photo-thermal conditions as described in the previous studies [2].

### Population structure, Genetic diversity and linkage disequilibrium

**Fig. 1:**
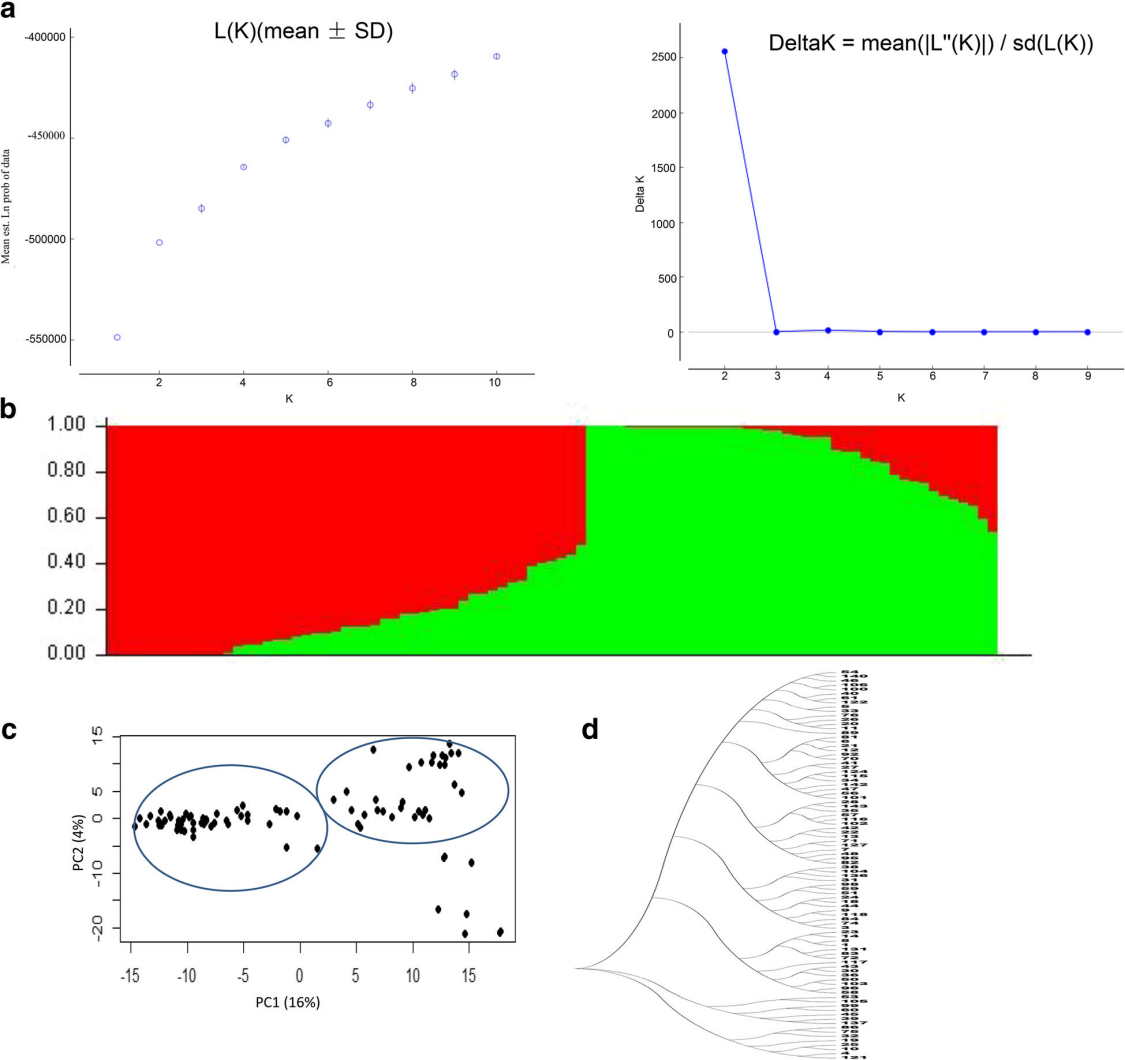
Population structure of 91 soybean cultivars using 5107 SNP markers. **a** Estimation of the number of sub-populations. The *left* figure was a plot of ln (probability of data) vs. K ranging from 1 to 10 and the *right* figure was a plot of subpopulation number vs. delta K values. **b** Population structure of 91 soybean cultivars based on SNP markers. The x-axis indicates the cultivars, and the y-axis indicates the Q value from STRUCTURE 2.3.1. The *red* color represents one sub-group, the *green* color represents another. **c** PCA of 91 soybean cultivars with the top two principal components. **d** Neighbor-joining tree of the 91 soybean cultivars

The population structure was assessed by STRUCTURE v.2.3.1 software based on SSR and SNP markers and the most likely number of sub-populations were consistent based on the two types of markers. When K = 2, the *ad hoc* quantity (ΔK) estimation had the highest value (Fig. 1a, Fig. 1b, Additional file 4: Figure S2a and Additional file 4: Figure S2b) [44]. The first sub-population contained 46 cultivars, a majority of which were from the late maturity groups in the Huang-Huai-Hai River Valley, and south China (95.7%). The cultivar ‘Altana’ from the US was also in this group. The second sub-population consisted of 45 cultivars of the early maturity groups (93.3%), which were from northeast China (60%) and the US (33.3%). A cluster analysis and PCA also showed that the genotypes were classified into two groups (Fig. 1c, Fig. 1d, Additional file 4: Figure S2c and Additional file 4: Figure S2d).

The averaged numbers of alleles per locus for SNPs and SSRs were 1.648 and 6.657, respectively, and the PIC values for SNPs and SSRs were 0.198 and 0.605, respectively (Table 2). The genetic diversity of SNP (0.250) is less than that of SSR (0.646), which is likely due to the difference of the bi-allele nature of SNP and the multi-allele nature of SSR. However, because the total number of SNPs is 29.7 times as high as that of SSR, indicating that SSR can provide more genetic information than SNP for assessment of genetic relatedness. The *Fst* between the two sub-populations defined by the STRUCTURE were 0.023 and 0.029 for SSRs and SNPs, respectively, which were similar to that between soybean breeding lines and landraces (0.0267) in a previous study [47]. Low population differentiation indicated a narrow genetic background in modern soybean cultivars.

**Table 2:** The genetic diversity of soybean population based on SSR and SNP markers

Marker	SNPs	SSRs
Major Allele Frquency	0.806	0.481
Alleles per locus	1.648	6.657
Gene Diversity	0.250	0.646
Heterozygosity	0.073	0.023
PIC	0.198	0.605
*Fst*	0.029	0.023

**Fig. 2:**
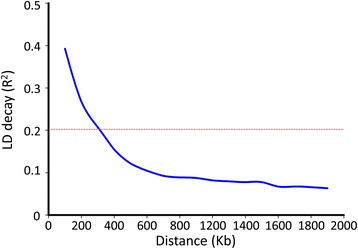
The estimated average linkage disequilibrium decay of soybean genome. The *dashed line* in *red* indicates the position where *r* ^2^ is 0.2

Linkage disequilibrium was analysed using SNPs with a minor allele frequency more than 5% and missing data less than 24%, the linkage disequilibrium of the population was decayed to *r* ^*2*^ = 0.2 within approximately 300 kb (Fig. 2). The result was consistent with the previous studies in soybean (125 kb -600 kb) [42].

### Genetic loci associated with flowering time under different photo-thermal conditions

A total of 118 SNPs with *p* < 9.79 × 10^−6^ and 11 SSRs with *p* < 2.86 × 10^−4^ were associated with the phenotypic values when GLM was performed (no loci was detected in 2009). The markers were further clumped based on the linkage disequilibrium blocks defined using the method described previously [48] and resulted in 87 QTLs for flowering time (Table 3). The proportion of genotypic variance explained by QTLs ranged from 13 to 35% among different environments (Table 3, Additional file 5: Figure S3, Additional file 6: Figure S4). The number of detected loci in each environment was different. There were 27, 23, 24, 24, 23, 45, 52 and 36 loci significantly associated with flowering in the LD + LT, LD + HT SP, SU, 14SP, 14SU, 15SP and 15SU, respectively. In addition, a total of 30 loci were detected in both pot experiments and field experiments, suggesting the soybean flowering were controlled by both environment-sensitive loci and environment-insensitive loci.

**Table 3:** The loci associated with flowering time and their phenotypic variation explained by the GLM model

Marker	Chr	Position	LD + LT	LD + HT	SP	SU	14SP	14SU	15SP	15SU	Known	Distance	Report QTLs^a^
											genes	(100Kbp)	
Gm01_53278791	Gm01	53278791						0.13	0.13				First flower 16-1
Gm01_53675540	Gm01	53675540	0.17										*N*
Gm02_10536842	Gm02	10536842						0.14	0.13	0.13			First flower 16–2
Gm02_11998056	Gm02	11998056	0.19										First flower 16–2 Pod maturity 19–1
Gm02_22829006	Gm02	22829006						0.16	0.15	0.16			First flower 13–4;
													First flower 13–2
Sat_135	Gm02	40366215			0.34	0.35							First flower 13–2
Gm03_1077329	Gm03	1077329								0.16			*N*
Gm03_5502496	Gm03	5502496		0.14				0.13	0.15				*N*
Gm03_36634361	Gm03	36634361			0.16					0.14			Pod maturity 16–4;
													Flower number 1–2
Gm03_38526701	Gm03	38526701					0.13						Flower number 1–2
Gm04_4497001	Gm04	4497001	0.16	0.16	0.15	0.17		0.13	0.16	0.13	*GmCOL3a*	2.78	First flower 22–1
Gm04_38840391	Gm04	38840391	0.16										*N*
Gm04_42951376	Gm04	42951376						0.14					Flower number 1–3
Gm04_46390533	Gm04	46390533							0.14				*N*
Gm05_682648	Gm05	682648								0.14			*N*
Gm05_1705841	Gm05	1705841		0.13									*N*
Gm05_26685967	Gm05	26685967						0.13	0.13				*N*
Gm05_38636402	Gm05	38636402		0.13				0.13	0.14				*N*
Gm05_40349605	Gm05	40349605						0.16	0.19	0.16			*N*
Gm06_2086304	Gm06	2086304			0.17								*N*
Gm06_2253042	Gm06	2253042			0.18	0.15							*N*
Satt422	Gm06	7227638							0.19				Pod maturity 26–1
Gm07_3143196	Gm07	3143196					0.13						First flower 4–2
Gm08_11052135	Gm08	11052135						0.13					Days to maturity 1-g1
Gm08_40882335	Gm08	40882335					0.17	0.14	0.15				*N*
Gm09_2327785	Gm09	2327785	0.16					0.15	0.15				*N*
Gm09_24238724	Gm09	24238724					0.13	0.14	0.13				First flower 3–4
Gm09_39822766	Gm09	39822766								0.14			Photoperiod insensitivity 5–2
Gm09_43508261	Gm09	43508261					0.13	0.17	0.19	0.13			*N*
Gm10_2317882	Gm10	2317882		0.14				0.15	0.14	0.15			Days to flowering 1-g18;
													Days to maturity 1-g14
Gm11_1161553	Gm11	1161553	0.17										*N*
Gm11_3950213	Gm11	3950213	0.15		0.14			0.14	0.14				Pod maturity 24–6
Gm11_4519147	Gm11	4519147						0.15	0.17				N
Gm11_5065170	Gm11	5065170	0.2	0.13					0.14	0.14			Node number 3–3
Gm11_6512939	Gm11	6512939						0.13					*N*
Gm11_6901726	Gm11	6901726	0.15										Flower number 1–5;
													Pod number 1–5
Satt197	Gm11	8879480							0.3	0.29			First flower 11–1;
													Pod maturity 17–1
Gm11_10847172	Gm11	10847172	0.18	0.14	0.21	0.21	0.23	0.27	0.28	0.23			Pod maturity 18–2
Gm11_11572077	Gm11	11572077			0.17	0.18	0.15	0.18	0.24	0.16			*N*
Gm11_16492046	Gm11	16492046	0.19	0.16	0.15	0.15			0.15				First flower 11–2;
													First flower 8–4
Gm11_17237725	Gm11	17237725	0.17				0.14	0.19	0.17	0.18			Pod maturity 18–1
Gm11_21023332	Gm11	21023332							0.15				First flower 11–2;
													First flower 8–4
Gm11_33034954	Gm11	33034954	0.17		0.19	0.21	0.13	0.25	0.26	0.24			*N*
Gm11_33555216	Gm11	33555216	0.18	0.15	0.15	0.19		0.17	0.19	0.18			*N*
Gm11_36174968	Gm11	36174968	0.16	0.14	0.16	0.16		0.15	0.17	0.18			Pod maturity 22–2
Gm12_5786241	Gm12	5786241			0.18	0.16							Reproductive stage length 7–3;
													maturity 26–2
Gm12_8435100	Gm12	8435100		0.14									*N*
Gm12_13354287	Gm12	13354287						0.13					*N*
Gm12_14231203	Gm12	14231203	0.15										*N*
Satt586	Gm13	11639980			0.19	0.2							First flower 11–4;
													Pod maturity 17–5
Gm13_23509779	Gm13	23509779						0.14	0.15	0.14			Photoperiod insensitivity 5–3
Gm13_39307253	Gm13	39307253	0.15								*GmCOL10b*	2.25	Days to flowering 1-g10
Gm14_7302299	Gm14	7302299	0.15	0.14				0.17	0.19	0.15			Flower number 1–6
Gm14_44697544	Gm14	44697544						0.15	0.15				*N*
Gm14_45457682	Gm14	45457682						0.14	0.14				First flower 21–1
Gm14_49107190	Gm14	49107190			0.14	0.15	0.14	0.27	0.21	0.19			First flower 21–1
Gm15_1265753	Gm15	1265753					0.17	0.15	0.14	0.13			First flower 12–3;
													Flower number 1–7
Gm15_13098003	Gm15	13098003		0.15		0.15	0.13	0.14	0.17	0.15			First flower 12–3;
													Flower number 1–7
Gm15_25411335	Gm15	25411335	0.17										*N*
Gm15_35867161	Gm15	35867161						0.16	0.15	0.16			*N*
Satt452	Gm15	38923152			0.17	0.2	0.18	0.18					*N*
Gm15_45004801	Gm15	45004801						0.15	0.15				*N*
Gm16_5773005	Gm16	5773005					0.14						*N*
SSRFT	Gm16	30741600			0.2	0.25	0.27	0.26	0.27	0.23	*GmFT2a*	inter-gene	GmFT2a
Gm16_30766209	Gm16	30766209	0.26	0.18	0.16	0.18	0.16	0.2	0.24	0.28	*GmFT2a;*	0.20;	First flower 9–3
											*GmFT2b*	0.14	
Gm16_35700223	Gm16	35700223	0.16	0.15	0.17	0.17	0.15	0.19	0.23	0.15			First flower 13–8;
													Photoperiod insensitivity 5–4
Gm17_37574384	Gm17	37574384							0.13				*N*
Gm17_41063513	Gm17	41063513							0.13				*N*
Gm18_4324818	Gm18	4324818	0.17	0.13									Pod maturity 22–9
Gm18_34401760	Gm18	34401760	0.18	0.17		0.16							Photoperiod insensitivity 2–2
Gm18_36929655	Gm18	36929655			0.18	0.2	0.14		0.14	0.16			First flower 10–2
Satt564	Gm18	47617795				0.21	0.26	0.21	0.24	0.22			Flower number 1–9;
													Pod number 1–8
Gm18_57126096	Gm18	57126096		0.13	0.15	0.14			0.14	0.14			*N*
Gm19_5195925	Gm19	5195925					0.13				*GmCOL2b*	2.85	*N*
Gm19_35449676	Gm19	35449676							0.14				*N*
Gm19_39723056	Gm19	39723056						0.14	0.14	0.14			First flower 15–2
Sat_113	Gm19	42110332				0.29	0.28	0.29	0.29	0.29			First flower 4–3;
													Pod maturity 24–10
Satt664	Gm19	46109700	0.26	0.19							*GmCOL11b*	0.14	Flower form 1–4b
Gm19_46761039	Gm19	46761039	0.16	0.15									First flower 13–9;
													Flower form 1–4;
													First flower 16–4
Satt229	Gm19	47049074			0.18								First flower 20–2;
													First flower 13–9;
													Flower form 1–4
Gm19_47514601	Gm19	47514601		0.14							*E3*	inter-gene	Flower form 1–4;
													First flower 16–4;
													First flower 5–2;
													First flower 5–3
Gm19_49786000	Gm19	49786000							0.15				First flower 5–3;
													First flower 8–3;
													First flower 16–4;
													Flower form 1–4
Satt571	Gm20	1291809	0.18										Pod maturity 24–5
Gm20_3880320	Gm20	3880320			0.22	0.2	0.17	0.16	0.18	0.18			First flower 16–3
Gm20_37857633	Gm20	37857633						0.14	0.15	0.14			First flower 16–3;
													Flower form 1–3
Gm20_43146832	Gm20	43146832	0.2	0.22	0.16	0.19	0.16	0.21	0.23	0.19	*GmCRY2c*	3.3	Flower number 1–11
Gm20_44260228	Gm20	44260228		0.13				0.14	0.14	0.15			Flower number 1–11

^a^QTLs are from http://www.soybase.org; N indicates that there were no reported QTL near the loci related to flowering time; Chr: chromosome; LD + LT: 16 h and spring sowingin 2010; LD + HT: 16 h and summer sowingin 2010. SP, Spring sowing season with natural day-length in 2010 pot experiment; SU, Summer sowing season with natural day-length in 2010 pot experiment; 14SP, Spring sowing in 2014 field experiment; 14SU, Summer sowing season in 2014 field experiment; 15SP, Spring sowing season in 2015 field experiment; 15SU, Summer sowing season in 2015 field experiment

A total of 32 markers were significantly associated with flowering time and were specific to photo-thermal (detected in only one environment) (Table 3). A total of 55 markers were associated with flowering time in two and more environments, among these, four markers (Gm11_10847171, Gm16_30766209, Gm16_35700223, Gm20_43146832) were identified in eight environments. The results indicated that these loci were important in controlling soybean flowering under multi-environments.

**Fig. 3:**
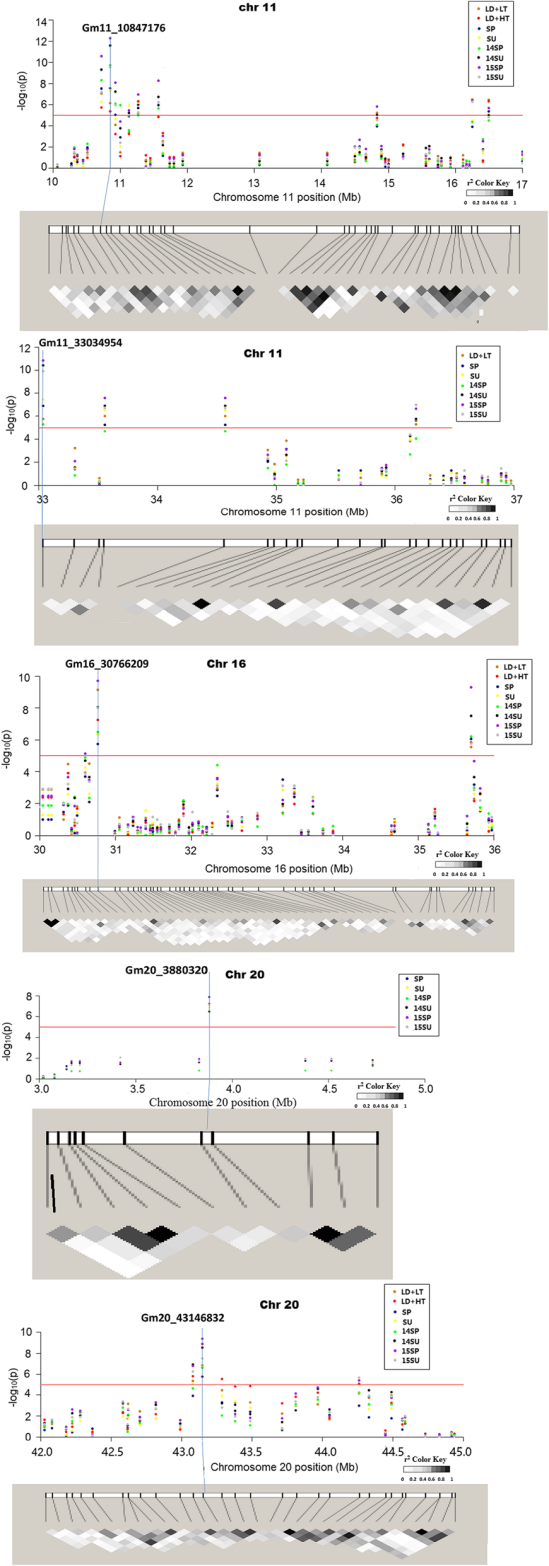
Manhattan plot and linkage disequilibrium block in different environments. Linkage disequilibrium blocks associated with flowering time near Gm11_10847172, Gm11_33034954, Gm16_30766209, Gm20_3880320 and Gm20_43146832. Significance threshold is denoted by the orange line. The up panel was the Manhattan plots of negative log10-transformed P values vs. SNPs. The down panel was haplotype block based on pairwise linkage disequilibriumr^2^values. LD, 16 h; LT, low temperature (spring sowing); HT, high temperature (summer sowing); SP, Spring sowing season with natural day-length in 2010 pot experiment; SU, Summer sowing season with natural day-length in 2010 pot experiment; 14SP, Spring sowing in 2014 field experiment; 14SU, Summer sowing season in 2014 field experiment; 15SP, Spring sowing season in 2015 field experiment; 15SU, Summer sowing season in 2015 field experiment

The most significant loci associated with flowering time varied under different photo-thermal conditions (Table 3, Fig. 3). Among SSR markers, Satt664 on Chr19 was the most significant locus associated with flowering time in the two LD conditions; Sat_135 on Chr02 was the most significant locus in the SP and SU conditions. Whereas, Sat_113 on Chr19 was the most significant locus in the 14SP, 14SU and 15SU conditions, respectively. Satt197 on Chr11 was the most significant locus with flowering time in the 15SP and 15SU conditions, respectively. Among SNPs, Gm11_10847172 was the locus most significantly associated with flowering time in the four conditions (SU, 14SP, 14SU and 15SP). Gm16_30766209 was the locus most significantly associated with flowering time in LD + LT and 15SU conditions, respectively. Gm20_43146832 on Chr20, Gm20_3880320 on Chr20 and Gm11_33034954 on Chr11 were the locus most significantly associated with flowering time in the LD + HT, SP and SU conditions, respectively. The significant SNPs were also detected in more than six environments, indicating that these loci may involve in regulation of flowering time in different photo-thermal conditions.

**Fig. 4:**
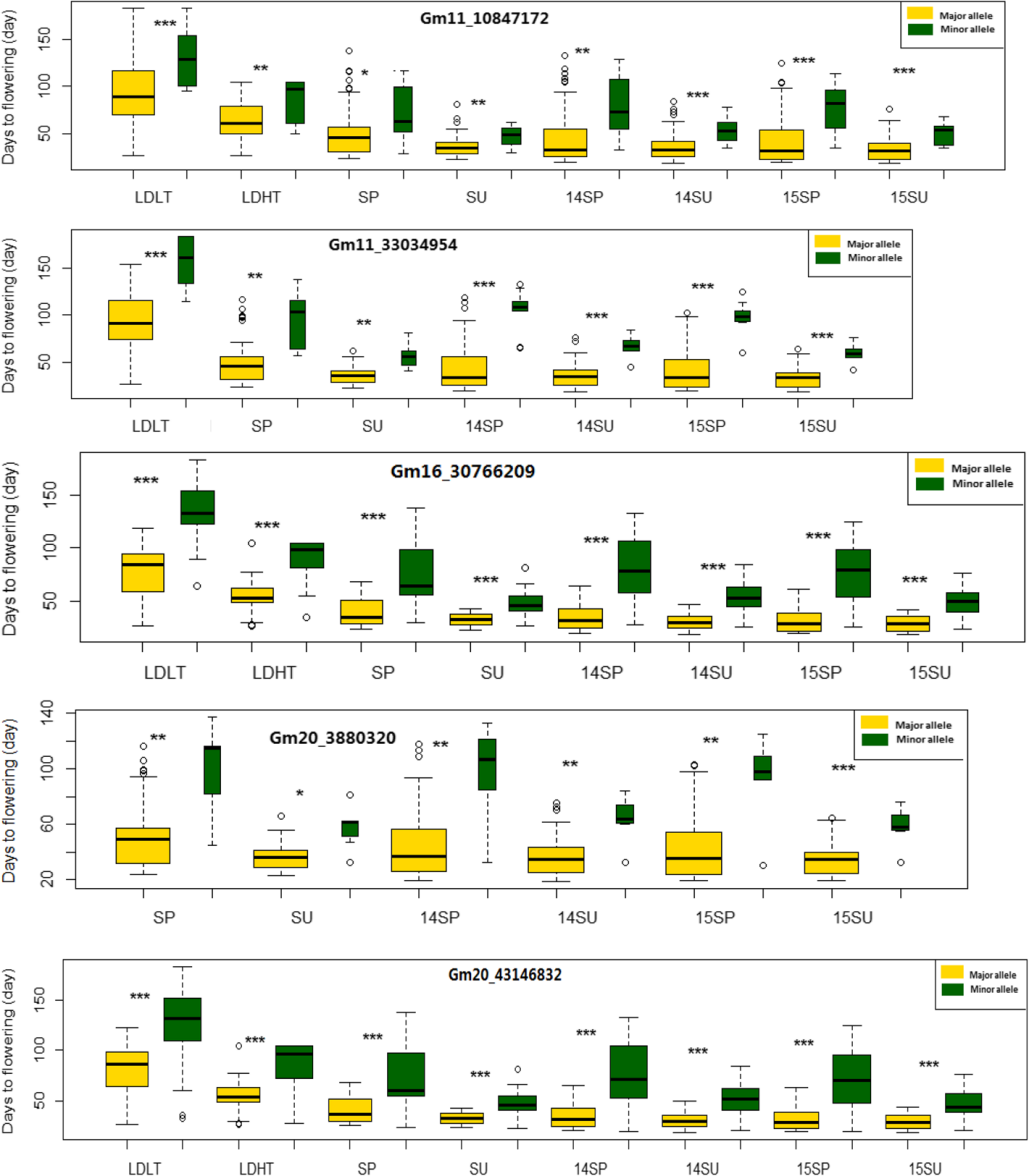
Phenotypic variation between cultivars carrying different alleles of the SNPs significantly associated with flowering time in various environments. The box plot shows the significant difference of days to flowering of the cultivars carrying two alleles of the SNPs. The significant SNPs were Gm11_10847172, Gm11_33034954, Gm16_30766209, Gm20_3880320 and Gm20_43146832. The major allele of significant loci was marked by *yellow*, and the minor allele was marked by *green*. Significant differences tested by the student’s *t*-test are also given (***p < 0.001, **p < 0.01, *p < 0.05). LD, 16 h; LT, low temperature (spring sowing); HT, high temperature (summer sowing); SP, Spring sowing season with natural day-length in 2010 pot experiment; SU, Summer sowing season with natural day-length in 2010 pot experiment; 14SP, Spring sowing in 2014 field experiment; 14SU, Summer sowing season in 2014 field experiment; 15SP, Spring sowing season in 2015 field experiment; 15SU, Summer sowing season in 2015 field experiment

The alleles of the significant SNPs (Gm11_10847172, Gm11_33034954, Gm16_30766209, Gm20_3880320 and Gm20_43146832) had different effects on flowering time across different photo-thermal conditions (Fig. 4, Additional file 7: Table S3). 53 and 38 cultivars contained T allele and C allele for the SNP Gm11_10847172, respectively. The flowering time of the cultivars with minor allele C were delayed for 38.5 d, 19.8 d, 21.8 d, 10.6 d, 32.4 d, 17.2 d, 33.4 d and 15.9 d compared with that of the cultivars with T allele (major allele) under the LD + LT, LD + HT, SP, SU, 14SP, 14SU, 15SP and 15SU, respectively. Similarly, the cultivars carrying the minor allele G of the SNP Gm20_43146832 were 48.7 d, 32.8 d, 31 d, 14.5 d, 39.4 d, 20.3 d, 37.4 d and 16.8 d later in flowering time than the those carrying the major allele A in the LD + LT, LD + HT, SP, SU, 14SP, 14SU, 15SP and 15SU conditions, respectively. The same patterns of the association of the two alleles with flowering time were also observed at other three significant loci Gm11_33034954, Gm16_30766209 and Gm20_3880320. Generally, LD could extend the difference of the flowering time between the cultivars carrying different alleles, while high temperature (summer sowing) could reduce the difference of the flowering time between the cultivars (Fig. 4, Additional file 7: Table S3).

### Genotype and environment interaction on soybean flowering time

To explore the genotype and environment interaction on soybean flowering time, we used the phenotype in 2010. The heritability of flowering time was 77.78%, and the heritability of additive and epistasis effects were 12.79% and 15.66%, respectively. The heritability of genotype × environment interaction was 49.33%, which was constituted by epistasis × environment interaction (h^2^_ae_ = 25.81%) and additive × environment interaction (h^2^_aae_ = 23.52%). These results indicated that soybean flowering time was mainly controlled by additive × environment interaction and the epistasis × environment interaction (Table 4).

**Table 4:** Estimated heritability of the flowering time in soybean

Total Heritability (%)	h^2^_a_ (%)	h^2^_ae_ (%)	h^2^_aa_ (%)	h^2^_aae_ (%)
77.78	12.79	23.52	15.66	25.81

a, additive effect; ae, additive by environment interaction effect; aa, epistasis effect; aae, epistasis by environment interaction effect; h^2^(%) = heritability(%)

**Fig. 5:**
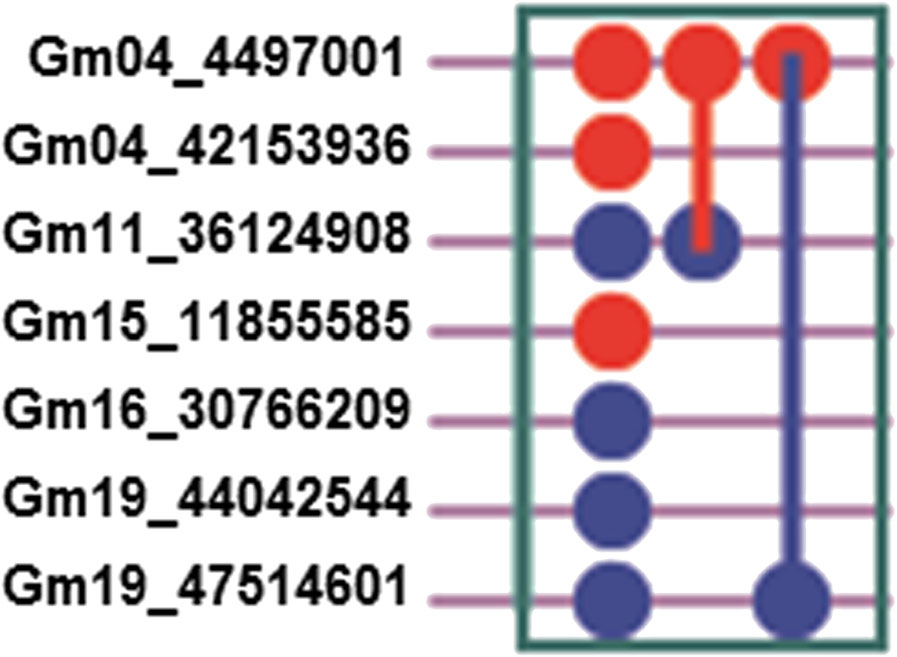
The plot of network of highly significant loci identified for soybean flowering time. The *red dots* represent the loci with additive effects; the *blue dots* represent the loci with both additive and environment-specific effects; red lines between two *dots* represent epistasis (aa); *blue lines* between two *dots* represent both epistasis (aa) and environment-specific epistasis (aae)

There were 7 loci with significant additive effects and/or additive × environment interaction effects, and 2 pairs of loci with significant epistatic effect and/or epistasis × environment interaction effects on soybean flowering time in six environments (Table 5, Fig. 5, Additional file 8: Figure S5). Gm04_4497001, Gm04_42153936 and Gm15_11855585 had significant additive effect, indicating that the additive loci were stable in different environments, whereas Gm11_36124908, Gm16_30766209, Gm19_44042544 and Gm19_47514601 had both significant additive effects and additive × environment interactions, suggesting that these loci were sensitive to different environments. Among them, Gm11_36124908 was the most significant and had high heritability of additive effect (h_a_^2^ = 6.73%) and additive × environment interaction (h_ae_^2^ = 31.96%). In addition, Gm04_4497001 interacted with two other loci (Gm11_36124908, Gm19_47514601) to control phenotypic variation of flowering time, and Gm04_4497001 and Gm19_47514601 had epistasis × environment interaction in the SP condition.

**Table 5:** The predicted genetic effects with significant heritability of the flowering time for soybeans in six environments

Locus	Effect	Predicted value	-Log_10_P	Heritability (%)	Candidate Genes
Gm04_4497001	*a*	−2.14	5.82	0.33	*Glyma04g06100*
Gm04_42153936	*a*	3.66	15.54	0.97	*Glyma04g358100; Glyma04g35720*
Gm11_36124908	*a*	−9.62	44.81	6.73	*Glyma11g34250*
	*ae1*	8.30	6.46	7.99	
	*ae2*	−6.01	3.66	7.99	
	*ae3*	−10.14	9.33	7.99	
	*ae4*	8.36	6.54	7.99	
Gm15_11855585	*a*	−2.65	8.61	0.51	*Glyma15g15730; Glyma15g15400*
Gm16_30766209	*a*	−5.00	11.79	1.81	*Glyma16g26660; Glyma16g26690*
	*ae1*	5.91	3.28	8.61	
	*ae3*	−14.26	16.21	8.61	
	*ae4*	6.29	3.65	8.61	
Gm19_44042544	*a*	4.71	25.36	1.61	Glyma19g36830
	*ae1*	−4.04	3.84	3.07	
	*ae3*	8.50	14.74	3.07	
	*ae4*	−4.10	3.95	3.07	
Gm19_47514601	*a*	−3.36	13.34	0.82	*Glyma19g40980*
	*ae2*	−4.40	4.68	2.02	
Gm04_4497001 × Gm11_36124908	*aa*	3.78	7.59	12.43	
Gm04_4497001 × Gm19_47514601	*aa*	1.93	4.82	3.23	
	*aae2*	3.78	3.90	12.45	

a, additive effect; aa, epistasis effect; ae1, additive by environment interaction effect in 12 h day length in the spring sowing (SD + LT); ae2, additive by environment interaction effect in natural day treatment in the spring sowing (SP); ae3, additive by environment interaction effect in 16 h day length in the spring sowing (LD + LT); ae4, additive by environment interaction effect in 12 h day length in the summer sowing (SD + HT); aae2, epistasis by environment interaction effect in natural day treatment in the spring sowing (SP); -Log_10_P = minus log_10_(*P*-value); h^2^(%) = heritability(%)

We also found that the direction of additive × environment interaction effect on soybean flowering time is dependent on photoperiod, whereas the magnitude of additive × environment interaction effect is dependent on temperature (Table 5, Additional file 8: Figure S5). For instance, the additive by environment interaction of Gm19_44042544 had a negative effect in the SD condition but positive in the LD condition, showing that the locus could enhance flowering time in the SD condition but delay flowering time in the LD condition. In contrast, the additive by environment interaction of Gm16_30766209 and Gm11_36124908 were positive in the SD condition but negative in the LD condition, suggesting that these loci could delay flowering time in the SD condition and accelerate flowering time in the LD condition. In response to photoperiod, the locus Gm19_44042544 showed opposite effect on flowering time compared with Gm16_30766209 and Gm11_36124908. On the other hand, for Gm16_30766209 and Gm11_36124908, the magnitude of delaying effect on flowering time was larger in the HT condition than in the LT condition, and the effect of Gm19_44042544 on the delay of flowering was also larger in the HT condition than that of the LT condition. These results indicate that high temperature could enhance both the positive or negative effects on flowering time in the SD conditions.

## Discussion

### The effects of genetic loci on soybean flowering time are dependent on photo-thermal conditions

In the present study, a large variation of days to flowering was observed among different environments and 49.33% of total phenotypic variation was contributed by environmental and genetic interaction, indicating that photo-thermal conditions played an essential role in determining soybean flowering time in addition to the genetic effects. The photo-thermal treatments in the current study provided a good opportunity for dissecting for dissecting the effects of photoperiod and temperature on soybean flowering time.

The environmental effect on the genetic variation of soybean flowering time had not been well documented [49]. In our previous study, 71 of 91 cultivars originated from different latitudes in China were selected to analyse the effects of photoperiod and temperature and the interaction between photoperiod and temperature on flowering time [37]. The results enhanced the understanding of the photo-thermal effects on flowering time at the phenotypic level. However, the effects of loci related to flowering time across photo-thermal conditions were not reported.

In this study, the effects of flowering-time-related loci in different photo-thermal conditions have been evaluated. Some loci were detected in only one environment, others were in multiple environments. The number of loci and their associated effects varied across different photo-thermal conditions. Interestingly, none of the loci was associated with the flowering time in the SD treatment. In the previous *Arabidopsis* studies, there were few QTLs linked to flowering time of the plant grown in Sweden than Italian conditions. It was speculated that the Sweden condition may represent saturated vernalization conditions, which could reduce the variation in flowering time among genotypes and result in reducing or removing the expression of some genes [50]. Similarly, soybean is a typical short day crop, we speculate that short day may also normalize soybean flowering time and remove contribution of some genes. The phenotypic variance of cultivars from different maturity groups became small in SD condition. Short days could reduce the effect of the dominant alleles of each dominant *E* genes on delaying flowering and maturity time in soybean [31].

### Interaction between loci and environment for soybean flowering time

Further analysis of the QTL detected by QTXNetwork confirmed the genetic variation underlying soybean flowering time across different environments. The expression of flowering time genes was influenced by environmental conditions, which is consistent with the results on *Arabidopsis thaliana* [29]. Jia et al. (2014) identified gene × environment interaction of cotton yield traits via the software QTXNetwork and classified genetic loci into three types: constituted loci (having no interaction with the environment), environment-specific loci (detected only in one environment), and environment-sensitive loci (the effect of the loci being dependent upon the environment) [35]. Our study identified the same types of loci with both additive and epistatic effects, and their interactions with the environment that controlled soybean flowering time. Our result is inconsistent with previous finding that soybean flowering time is mainly controlled by the additive effect [20]. This inconsistency may result from different genetic backgrounds of materials used in different studies. Previous evidence showed that epistasis played an important role in controlling flowering time, and epistasis explained a portion of the ‘missing heritability’ in plants [51]. In *Arabidopsis*, phytochrome A (PhyA) interacts with CO protein in the photoperiod pathway, and *CO* interacts with gibberellins to regulate the expression of *FT* in the GA pathway [52]. Gm04_4497001 (*CO*) identified in the present study may be a core locus of epistasis interacting with other loci for controlling soybean flowering time. In our previous studies on soybean photo-thermal responses, we proposed that photoperiod determines whether soybean plant is reproductive or vegetative, whereas temperature controls its developmental rate, and the magnitude of temperature effects depends upon the developmental status of the plants (reproductive or vegetative) [53, 54]. Through the analysis of the interaction between genotypes and environments in the current study, we found that whether the additive × environment interaction effect on soybean flowering time was positive or negative was dependent on photoperiod, whereas the magnitude of additive × environment interaction effect was on temperature, which is consistent with the model of photo-thermal interactions on flowering time in soybean [53, 54].

### The flowering time loci and candidate genes

In this study, SSR markers were mainly selected based on the previous linkage analysis related to important agronomic traits, particularly phenological traits. Nine of the 11 significant SSR markers found in this study were previously reported to be linked to flowering time and maturity. Several SNPs identified in the present study were located in or adjacent to the previously reported QTLs (Table 2). Two clusters of significant markers in Gm11 (10 Mb-17 Mb) and Gm11 (33 Mb-36 Mb) were significantly associated with flowering time. Gm11 (10 Mb-17 Mb) contained two flowering time related QTLs [55, 56] and two maturity QTLs [57], this region was also reported to be linked to flowering time in an association population [58]. The cluster of significant markers on Gm19 (46 Mb-48 Mb) was consistently identified to be closely linked to soybean flowering time through linkage mapping and related to maturity and plant height through association mapping [59] (Table 3). The cluster of significant markers on Gm20 (43 Mb-44 Mb) identified the same genomic region of flower number QTLs. The markers in those regions could potentially be used by soybean breeders to improve soybean adaptability. Additionally, 35 novel loci associated with soybean flowering time were identified.

**Fig. 6:**
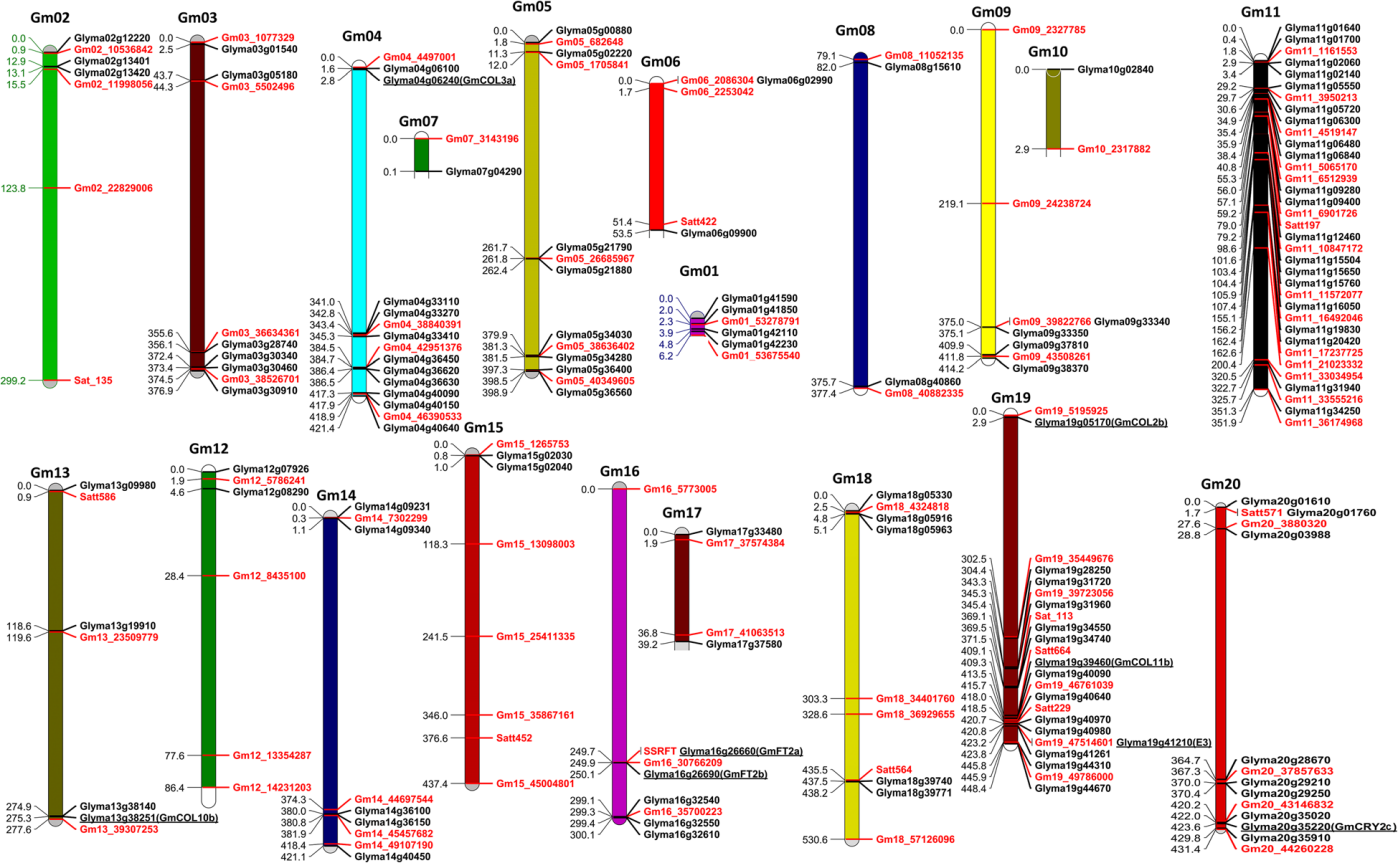
The positions of flowering time-related loci and their corresponding candidate genes. The positions of candidate genes were marked in *black*, the loci were shown in red,  and the known flowering genes were underlined. The position of the first locus on each chromosome was set as zero, and the *left* number showed the relative in the genome, 1 = 100 kb

Identification of genes involved in soybean flowering time may give us a better understanding of the genetic mechanism underlying the environmental regulation on soybean flowering time (Table 3, Fig. 6, Additional file 9: Table S4, Additional file 10: Table S5). The loci Gm04_4497001, Gm16_30766209 and Gm19_47514601 were identified to be associated with flowering time using both TASSEL and QTXNetwork software. Of the four important flowering genes *Glyma04g06240* (*GmCOL3a*), *Glyma16g26660* (*GmFT2a*), *Glyma16g26690* (*GmFT2b*) and *Glyma19g41210* (*E3* or *GmPhyA3*) which were within 300 kb of the significant SNPs, *Glyma04g06240* (*GmCOL3a*) is located at 277.4 kb downstream of the peak SNP Gm04_4497001. *CONSTANS* (*CO*) is the key transcriptional activator of the gene that encodes the “florigen” protein FLOWERING LOCUS T (FT) in *Arabidopsis* [60]. *Glyma16g26660* and *Glyma16g26690* were close to the significant SNP Gm16_30766209, with physical distances of 19.9 kb and 14.3 kb, respectively. *Glyma16g26660* and *Glyma16g26690* are the key flowering time genes *GmFT2a* and *GmFT2b*, and *GmFT2a* is identified as the key flowering integrator in soybean [24]. Gm19_47514601 is located between exon 2 and exon 3 of *Glyma19g41210* (*E3* or *GmPhyA3*), which encodes the phytochrome A (PHYA) protein [13], a far-red receptor involved in stabilizing the flowering activator CONSTANS (CO) protein during the late afternoon [61]. The peak SNP, Gm20_3880320, detected in the SP condition was located 61.6 kb upstream of the gene *Glyma20g03988*, a homolog of *PFT1* (phytochrome and flowering time regulatory protein 1) in *Arabidopsis*, which was an activator of flowering in a photoperiod pathway [62]. In the LD + HT condition, the peak SNP, Gm20_43146832, is 169.2 kb upstream of the gene *Glyma20g35020*, a homologous gene encoding COP1-interacting protein, which is a regulator of light-regulated genes and a potential direct downstream target of *COP1* for mediating light control of gene expression [63]. Gm11_33034954 was the peak SNP in SU conditions, and 215.2 kb upstream of the flower gene *Glyma11g31940*, which was predicted to encode auxin response factor 8. The peak SNP, Gm11_10847172, detected in the SU, 14SP, 14SU and 15SP four conditions was located 294.25 Kb upstream of the gene *Glyma11g15504*, a homolog of CONSTANS protein, which has not been reported in soybean. These results indicate that our methods of association mapping and genetic effect analysis across different photo-thermal conditions were efficient in detecting the major and significant genomic regions (QTL) and genes regulating soybean flowering time. The markers associated with these loci can be utilized as markers for marker-assisted breeding for improving soybean adaptation.

### The implication of loci associated with flowering time for soybean adaptation improvement

The photo-thermal treatments in the current study were designed to simulate the natural conditions in three main soybean production regions in China, so the results could facilitate soybean breeding in those regions. The treatment of long day-length and spring-sowing in the current study is similar to the growth conditions in the northeast spring-sowing region, whereas the short day-length with spring-sowing and summer-sowing treatments resemble with the growth conditions in the south spring-sowing and south summer-sowing regions. The natural day-length with different sowing seasons in Beijing simulates the growth conditions of spring and summer-sowing soybeans in the Huang-Huai-Hai River Valley. The peak locus on Gm19 (Satt664) under the LD + LT treatment is a useful marker for marker-assisted selection of adaptation in the northeast China, whereas the loci Sat_135, Gm11_10847172, Gm11_33034954, and Gm20_3880320, could be utilized for selection in the Huang-Huai-Hai River Valley. The markers, Gm16_30766209 and Gm11_36124908, detected in both the LD and SD conditions could be utilized for selection in both northeast and south China.

## Conclusions

In this study, a total of 87 markers (11 SSRs and 76 SNPs) associated with flowering time of soybean were identified via GWAS. The number and effect of loci associated with flowering time of soybean depended on the photo-thermal conditions. The loci with large effects were found to be located on Gm 11, Gm 16 and Gm 20, consistent with previous reports. The variation of soybean flowering time among the cultivars mainly resulted from gene × environment interactions, particularly epistasis × environment interaction and additive × environment interaction. Gm04_4497001 (close to *GmCOL3a*), Gm16_307609 (close to *GmFT2a* and *GmFT2b*), and Gm19_47514601 (close to *E3* or *GmPhyA3*) are important for controlling flowering time. Among them, Gm04_4497001 may be the major locus with epistatic interaction with other loci for controlling flowering time. The direction and magnitude of the interaction between loci and environments were dependent on photo-thermal conditions, indicating that photoperiod determines the developmental status of plant (vegetative or vegetative), but temperature controls the developmental rate of plant. In summary, the results provide insights into the genetic basis of soybean flowering time and markers could be used for marker-assisted breeding to improve soybean adaptation.

## Additional files

Additional file 1: Table S1. The origin, ecotypes and maturity groups of the soybean cultivars in this study. (DOCX 27 kb)

Additional file 2: Table S2. Polymorphic SSR and SNP markers used for this study. (XLSX 230 kb)

Additional file 3: Figure S1. The histogram of soybean flowering time in each environment. (a) The histogram of soybean flowering time in 2009. (b) The histogram of soybean flowering time in 2010. (c) The histogram of soybean flowering time in 2014 and 2015, respectively. (DOCX 5435 kb)

Additional file 4: Figure S2. Population structure of 91 soybean cultivars using 63 SSR markers. (a) Estimation of the number of sub-populations. The left figure was a plot of ln (probability of data) vs. K ranging from 1 to 12 and the right figure was a plot of subpopulation number vs. delta K values. (b) Population structure of 91 soybean cultivars based on 63 SSR markers. The x-axis indicates the cultivars, and the y-axis indicates the Q value from STRUCTURE 2.3.1. The red color represents one sub-group, the green color represents another. (c) PCA of 91 soybean cultivars with the top two principal components. (d) Neighbor-joining tree of the 91 soybean cultivars. (DOCX 498 kb)

Additional file 5: Figure S3. Genome-wide association scan for flowering time in different environments using SNPs. (a) The Quantile-Quantile Plot; (b) Manhattan plot for days to flowering. *P*-values (negative log-transformed) are shown in the plot relative to their position on each of the 20 chromosomes. The horizontal pink line indicates the genome-wide significant threshold (9.79 × 10^−6^). (DOCX 469 kb)

Additional file 6: Figure S4. Manhattan plot for days to flowering in the association panel in different environments using SSRs. (a) Quantile-Quantile Plot (b) Manhattan plot for days to flowering. *P*-values (negative log-transformed) are shown in the plot relative to their genetic positions, the horizontal pink line indicates the genome-wide significant threshold (2.86 × 10^−4^). (DOCX 384 kb)

Additional file 7: Table S3. The mean flowering time of the accession carrying different alleles. (DOCX 21 kb)

Additional file 8: Figure S5. The plot of the interactions between significant loci with the flowering time and environment detected by the QTXNetwork. Red columns represent general QTX effects for all six environments. The green lines denote the n-th environment-specific effect. 1, SD + LT condition; 2, SP condition; 3, LD + LT condition; 4, SD + HT condition; 4–42, Gm04_4497001; 4–154, Gm04_42153936; 11–190, Gm11_36124908; 15–116, Gm15_11855585; 16–152, Gm16_30766209; 19–208, Gm19_44042544; 19–243, Gm19_47514601. (DOCX 407 kb)

Additional file 9: Table S4. The significant loci associated with flowering time and related candidate genes. (DOCX 27 kb)

Additional file 10: Table S5. The position of the loci and the corresponding candidate genes. (XLSX 18 kb)

## Abbreviations

Chr: Chromosome
cM: Centi Morgan
E: Environment
G: Genetic composition
G × E: The interaction between genotype and environment
GLM: General linear model
GWAS: Genome-wide association study
HT: High temperature
ILD: Incandescent lamps
LD: 16 h day length
LG: Linkage group
LT: Low temperature
MG: Maturity group
NILs: Near isogenic lines
QTL: Quantitative trait loci
RIL: Recombinant inbred lines
SD: 12 h day length
SNP: Single nucleotide polymorphism
SP: Spring sowing season with natural day-length in Beijing
SSR: Simple sequence repeat
SU: Summer sowing season with natural day-length in Beijing
VC: Growth stage where cotyledons are fully developed
