# Supplementary material for: Association mapping of loci controlling genetic and environmental interaction of soybean flowering time under various photo-thermal conditions
Source: BMC Genomics. 2017 May 26;18:415. doi: 10.1186/s12864-017-3778-3 (PMC5446728; doi:10.1186/s12864-017-3778-3)
Supplement: Supplementary file 1 — The origin, ecotypes and maturity groups of the soybean cultivars in this study. (DOCX 27 kb) [file 12864_2017_3778_MOESM1_ESM.docx]

**TableS1The origin, ecotypes and maturity groups of the soybean cultivars tested in this study**

| Cultivar | Origin | Ecotype | Putative Maturity Group^a^ | The test cultivars in 2009 |
| --- | --- | --- | --- | --- |
| Beihudou | Beian, Heilongjiang, China | Nsp | MG000 | * |
| Heihe3 | Aihui, Heilongjiang, China | Nsp | MG00 |  |
| Heihe54 | Aihui, Heilongjiang, China | Nsp | MG00 |  |
| Fengshou10 | Keshan, Heilongjiang, China | Nsp | MG0 |  |
| Fengshou12 | Keshan, Heilongjiang, China | Nsp | MG0 | * |
| Hongfeng2 | Youyi, Heilongjiang, China | Nsp | MG0 | * |
| Dongnong72-806 | Harbin, Heilongjiang, China | Nsp | MG0 |  |
| Dongnong4 | Harbin, Heilongjiang, China | Nsp | MG0 |  |
| Heinong16 | Harbin, Heilongjiang, China | Nsp | MGⅠ | * |
| Heinong26 | Harbin, Heilongjiang, China | Nsp | MGⅠ |  |
| Jilin3 | Gongzhuling, Jilin, China | Nsp | MGⅠ |  |
| Jilin4 | Gongzhuling, Jilin, China | Nsp | MGⅠ | * |
| Jilin13 | Gongzhuling, Jilin, China | Nsp | MGⅠ |  |
| Zaofeng1 | Gongzhuling, Jilin, China | Nsp | MGⅠ |  |
| Jiunong9 | Jilin City, Jilin, China | Nsp | MGⅠ | * |
| Tiefeng18 | Tieling, Liaoning, China | Nsp | MGⅡ | * |
| Tiefeng19 | Tieling, Liaoning, China | Nsp | MGⅡ |  |
| Tiefeng20 | Tieling, Liaoning, China | Nsu | MGⅡ |  |
| Jin6606-24 | Jinzhou, Liaoning, China | Nsp | MGⅡ |  |
| Tiefeng8 | Tieling, Liaoning, China | Nsp | MGⅢ |  |
| Dandou2 | Fengcheng, Liaoning, China | Nsp | MGⅢ | * |
| Dandou4 | Fengcheng, Liaoning, China | Nsp | MGⅢ |  |
| Jin33 | Jinzhou, Liaoning, China | Nsp | MGⅢ | * |
| Jin8-14 | Jinzhou, Liaoning, China | Nsp | MGⅢ |  |
| Qunyingdou | Pingquan, Hebei, China | Hsp | MGⅡ |  |
| Jidou1 | Shijiazhuang, Hebei, China | Nsu | MGⅢ | * |
| Naiyinheidou | Qian’an, Hebei, China | Hsp | MGⅣ |  |
| Bahong1 | Baxian, Hebei, China | Nsp | MGⅣ |  |
| Huairouhuangdou | Huanrou, Beijing, China | Hsp | MGⅣ | * |
| Youbian30 | Beijing, China | Hsp | MGⅣ | * |
| Jindou1 | Taigu, Shanxi, China | Nsp | MGⅡ | * |
| Jindou3 | Taigu, Shanxi, China | Nsp | MGⅡ | * |
| Jindou2 | Taigu, Shanxi, China | Nsp | MGⅢ | * |
| Jinda814 | Taigu, Shanxi, China | Nsp | MGⅢ |  |
| Jindou4 | Taiyuan, Shanxi, China | Nsp | MGⅣ |  |
| Lyupidou | Fengxiang, Shanxi, China | Nsu | MGⅤ |  |
| Wandouzao | Lueyang, Shanxi, China | Ssp | MGVIII |  |
| Qihuang10 | Jinan, Shandong, China | Nsu | MGⅢ |  |
| Fengshouhuang | Weifang, Shandong, China | Nsu | MGⅣ |  |
| Yuejin4 | Heze, Shandong, China | Nsu | MGⅣ | * |
| Zhengzhou135 | Zhengzhou, Henan, China | Nsu | MGⅢ | * |
| Chenliuniumaohuang | Chengliu, Henan, China | Nsu | MGⅣ |  |
| Zidadou | Zhumadian, Henan, China | Nsu | MGⅤ |  |
| Taixingheidou | Taixing, Jiangsu, China | Ssp | MG0 |  |
| Xudou1 | Xuzhou, Jiangsu, China | Nsu | MGⅣ |  |
| Xudou2 | Xuzhou, Jiangsu, China | Nsu | MGⅣ | * |
| Xuzhou5 | Xuzhou, Jiangsu, China | Nsu | MGⅣ |  |
| 58-161 | Binghai, Jiangsu, China | Nsu | MGⅤ |  |
| Nannong493-1 | Nanjing, Jiangsu, China | Ssu | MGVII | * |
| Shangyukanshanbai | Shangyu, Zhejiang, China | Ssp | MGⅢ |  |
| Lanxidaqingdou | Lanxi, Zhejiang, China | Sau | MGVIII |  |
| Fengxiansuidaohuang | Fengxian, Shanghai, China | Ssu | MGⅥ |  |
| Aijiaozao | Wuhan, Hubei, China | Ssp | MGII | * |
| Houzimao | Wuhan, Hubei, China | Ssu | MGⅥ |  |
| Edou2 | Wuhan, Hubei, China | Ssu | MGⅥ |  |
| Mianyanghuangfengwo | Mianyang, Hubei, China | Ssu | MGVII |  |
| Liuyuezao | Guanlin, Guizhou, China | Ssp | MGⅣ |  |
| Liuyuehuang | Liuzhi, Guizhou, China | Ssp | MGⅣ |  |
| Baishuidou | Anshun, Guizhou, China | Ssp | MGⅤ |  |
| Yishanliuyuehuang | Yishan, Guangxi, China | Ssp | MGⅤ | * |
| Yulindahuangdou | Yulin, Guangxi, China | Ssp | MGVII | * |
| Pingguohuangdou | Pingguo, Guangxi, China | Ssu | MGVIII |  |
| Heibiqing | Gaozhou, Guangdong, China | Ssp | MGⅢ | * |
| Yangchunqingdou | Yangchun, Guangdong, China | Sau | MGⅥ |  |
| Baihuadou | Gaozhou, Guangdong, China | Ssp | MGⅤ |  |
| Jinjiangdaqingren | Jinjiang, Fujian, China | Ssp | MGⅤ |  |
| Lianchengbaihuadou | Liancheng, Fujian, China | Sau | MGVIII |  |
| Jiangledaqingdou | Jiangle, Fujian, China | Sau | MGVIII |  |
| Bayueqingdou | Hengyang, Hunan, China | Ssu | MGVIII |  |
| Qiudou1 | Hengyang, Hunan, China | Sau | MGVIII | * |
| Fengchengchadou | Fengcheng, Jiangxi, China | Ssp | MGⅢ | * |
| Ruijingxiaohuangdou | Ruijin, Jiangxi, China | Sau | MGVII |  |
| Shangraodaqingsi | Shangrao, Jiangxi, China | Sau | MGVII |  |
| Aijiaoqing | Xinyu, Jiangxi, China | Sau | MGVIII |  |
| Zigondongdou | Zigong, Sichuan, China | Ssu | MGVIII |  |
| Merit | USA |  | MG0 |  |
| Portage | USA |  | MG0 |  |
| Altona | USA |  | MG0 |  |
| Clay | USA |  | MG0 |  |
| Chippewa 64 | USA |  | MGI |  |
| Hacker | USA |  | MGI |  |
| Corsoy | USA |  | MGII |  |
| Amsoy 71 | USA |  | MGII |  |
| Beeson | USA |  | MGⅡ |  |
| Harosoy 63 | USA |  | MGⅢ |  |
| Clark | USA |  | MGIV |  |
| Karlet | USA |  | MGIV |  |
| Mike | USA |  | MGV |  |
| Hood | USA |  | MGVI |  |
| Wayne | USA |  | MGVI |  |
| Dumn | USA |  | unknown |  |

^a^Thematurity groups of cultivars from China were determined based on the data of previous ecological experiments[[1](#_ENREF_1)]. * The cultivars tested in 2009; Nsp, Northern spring sowing type; Hsp, Huang-Huai-Hai spring type; Hsu, Huang-Huai-Hai summer sowing type; Ssp, Spring sowing type in south China; Ssu, summer sowing type in south China; Sau, autumn sowing type in south China.

**Reference**

1. Hao G, Chen X, BuM: **Classification of the chinese soybean cultivars into maturity groups.***Acta AgrinimicaSinica* 1992, **18**:275-281.
