# Supplementary material for: Association mapping of loci controlling genetic and environmental interaction of soybean flowering time under various photo-thermal conditions
Source: BMC Genomics. 2017 May 26;18:415. doi: 10.1186/s12864-017-3778-3 (PMC5446728; doi:10.1186/s12864-017-3778-3)
Supplement: Supplementary file 3 — The histogram of soybean flowering time in each environment. (a) The histogram of soybean flowering time in 2009. (b) The histogram of soybean flowering time in 2010. (c) The histogram of soybean flowering time in 2014 and 2015, respectively. (DOCX 5435 kb) [file 12864_2017_3778_MOESM3_ESM.docx]

**
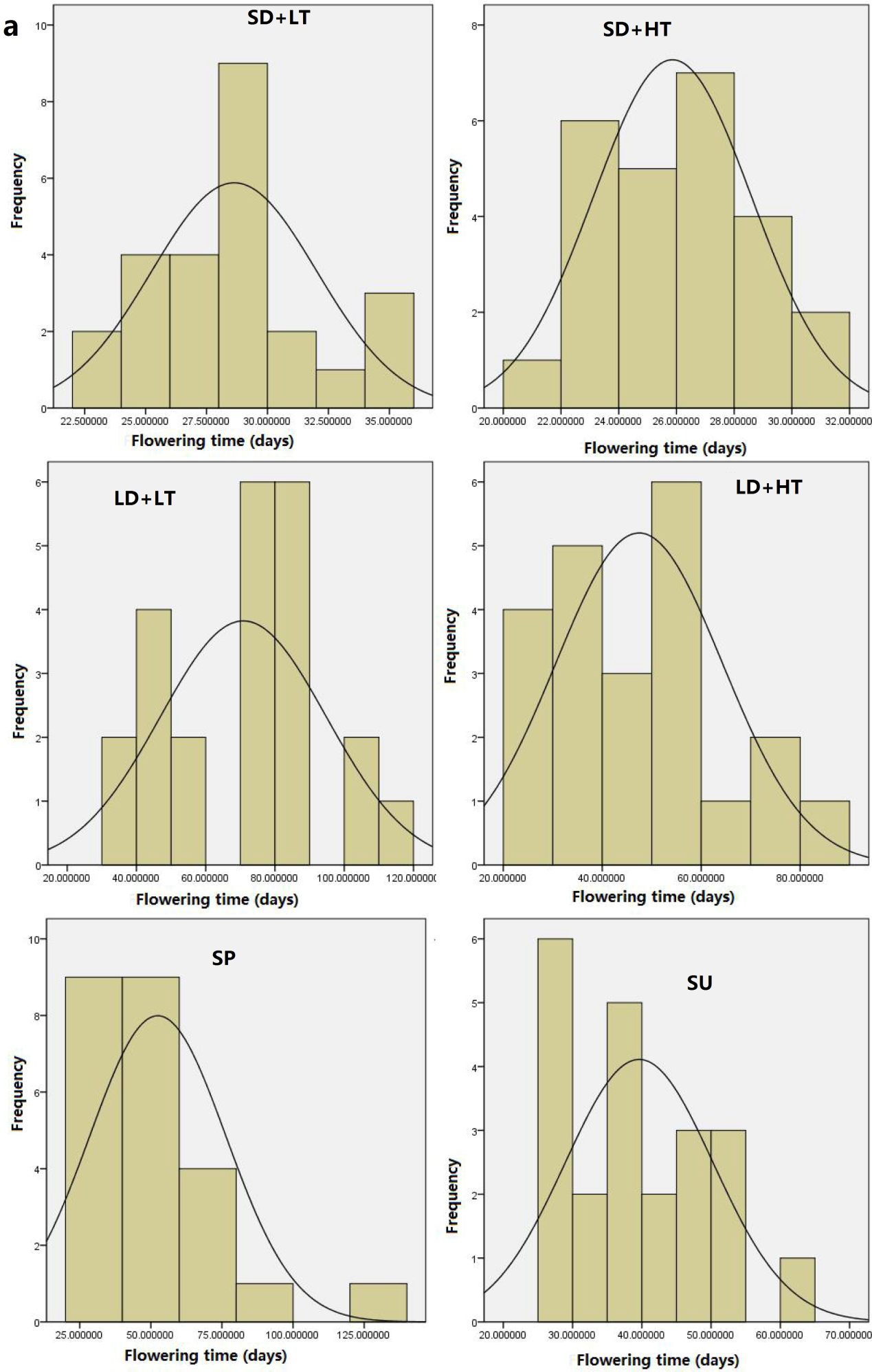
**

**
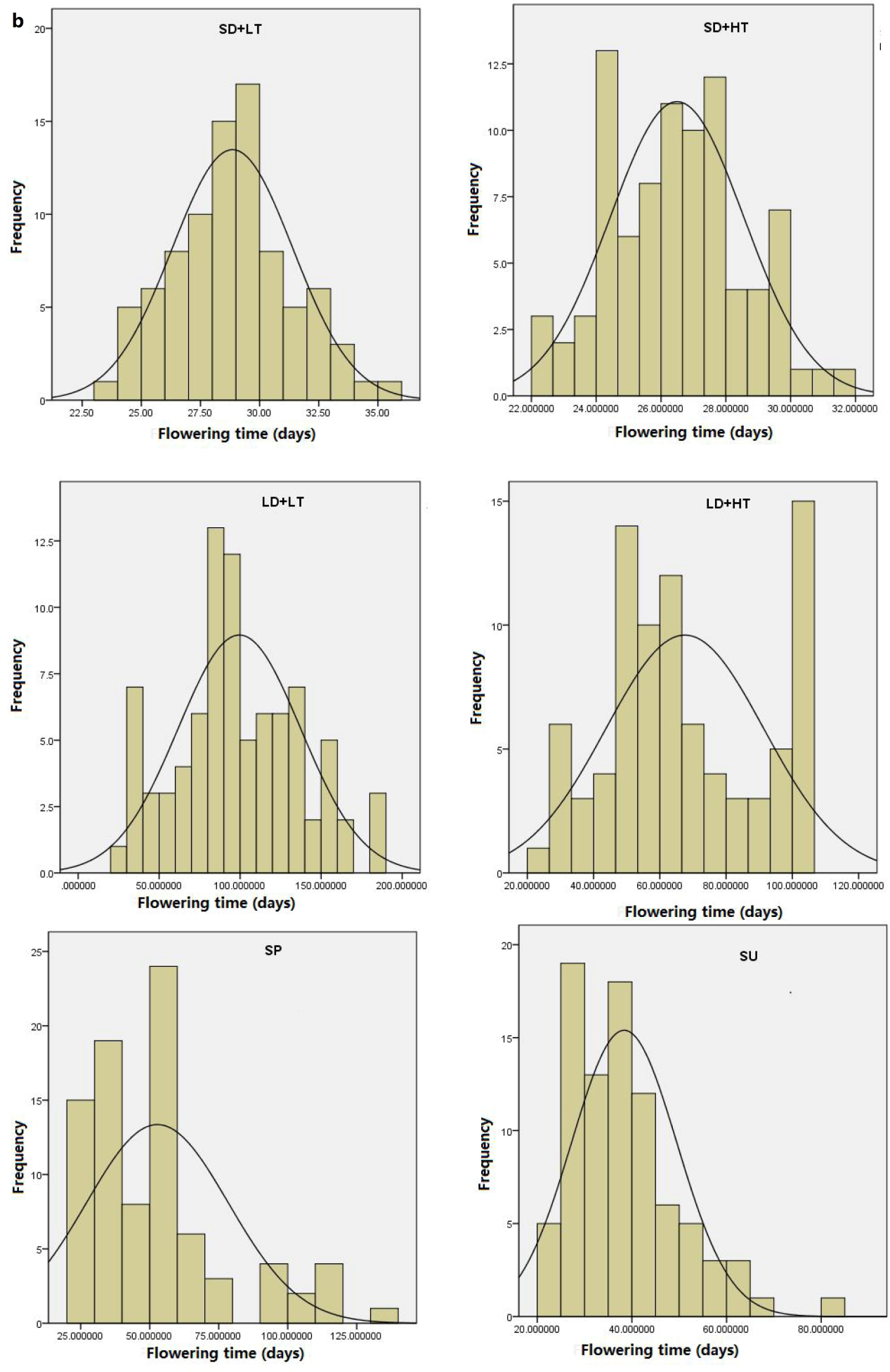
**

**
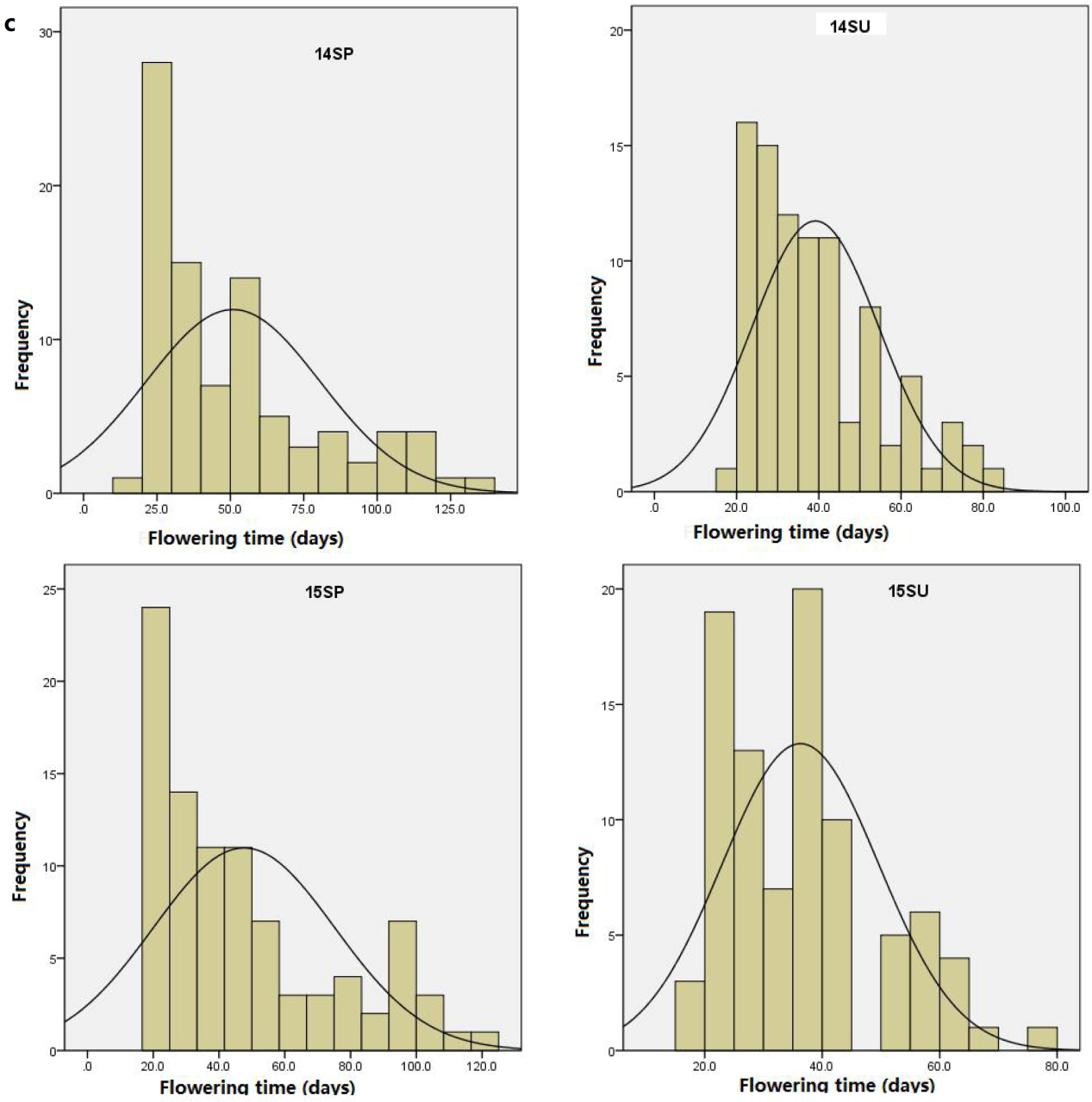
**

**Figure S1 The histogram of soybean flowering time in each environment. (a) The histogram of soybean flowering time in 2009. (b) The histogram of soybean flowering time in 2010. (c) The histogram of soybean flowering time in 2014 and 2015.**

SD, 12 h; LD, 16 h; LT, low temperature (spring sowing); HT, high temperature (summer sowing); SP, Spring sowing season; SU, Summer sowing season; 14SP, Spring sowing season in 2014; 14SU, Summer sowing season in 2014; 15SP, Spring sowing season in 2015; 15SU, Summer sowing season in 2015.
