# Supplementary material for: Association mapping of loci controlling genetic and environmental interaction of soybean flowering time under various photo-thermal conditions
Source: BMC Genomics. 2017 May 26;18:415. doi: 10.1186/s12864-017-3778-3 (PMC5446728; doi:10.1186/s12864-017-3778-3)
Supplement: Supplementary file 4 — Population structure of 91 soybean cultivars using 63 SSR markers. (a) Estimation of the number of sub-populations. The left figure was a plot of ln (probability of data) vs. K ranging from 1 to 12 and the right figure was a plot of subpopulation number vs. delta K values. (b) Population structure of 91 soybean cultivars based on 63 SSR markers. The x-axis indicates the cultivars, and the y-axis indicates the Q value from STRUCTURE 2.3.1. The red color represents one sub-group, the green color represents another. (c) PCA of 91 soybean cultivars with the top two principal components. (d) Neighbor-joining tree of the 91 soybean cultivars. (DOCX 498 kb) [file 12864_2017_3778_MOESM4_ESM.docx]

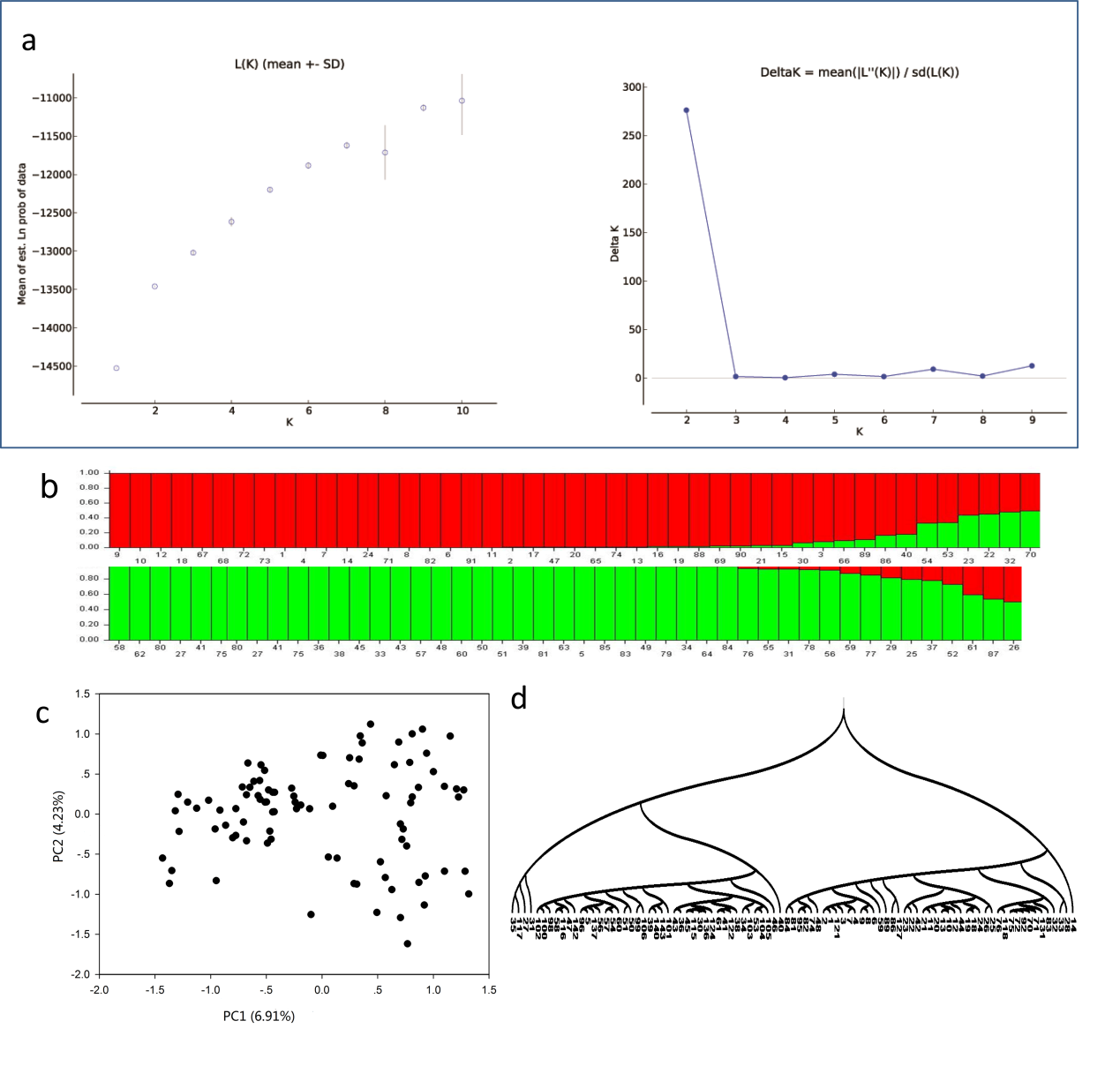


**Figure S2. Population structure of 91 soybean cultivars using 63 SSR markers. (a) Estimation of the number of sub-populations.** The left figure was a plot of ln (probability of data) vs. K ranging from 1 to 10 and the right figure was a plot of subpopulation number vs. delta K values. **(b) Population structure of 91 soybean cultivars based on 63 SSR markers.** The x-axis indicates the cultivars, and the y-axis indicates the Q value from STRUCTURE 2.3.1. The red color represents one sub-group, the green color represents another. **(c) PCA of 91 soybean cultivars with the top two principal components**. **(d) Neighbor-joining tree of the 91 soybean cultivars.**
