# Supplementary material for: Association mapping of loci controlling genetic and environmental interaction of soybean flowering time under various photo-thermal conditions
Source: BMC Genomics. 2017 May 26;18:415. doi: 10.1186/s12864-017-3778-3 (PMC5446728; doi:10.1186/s12864-017-3778-3)
Supplement: Supplementary file 5 — Genome-wide association scan for flowering time in different environments using SNPs. (a) The Quantile-Quantile Plot; (b) Manhattan plot for days to flowering. P-values (negative log-transformed) are shown in the plot relative to their position on each of the 20 chromosomes. The horizontal pink line indicates the genome-wide significant threshold (9.79 × 10^−6^). (DOCX 469 kb) [file 12864_2017_3778_MOESM5_ESM.docx]

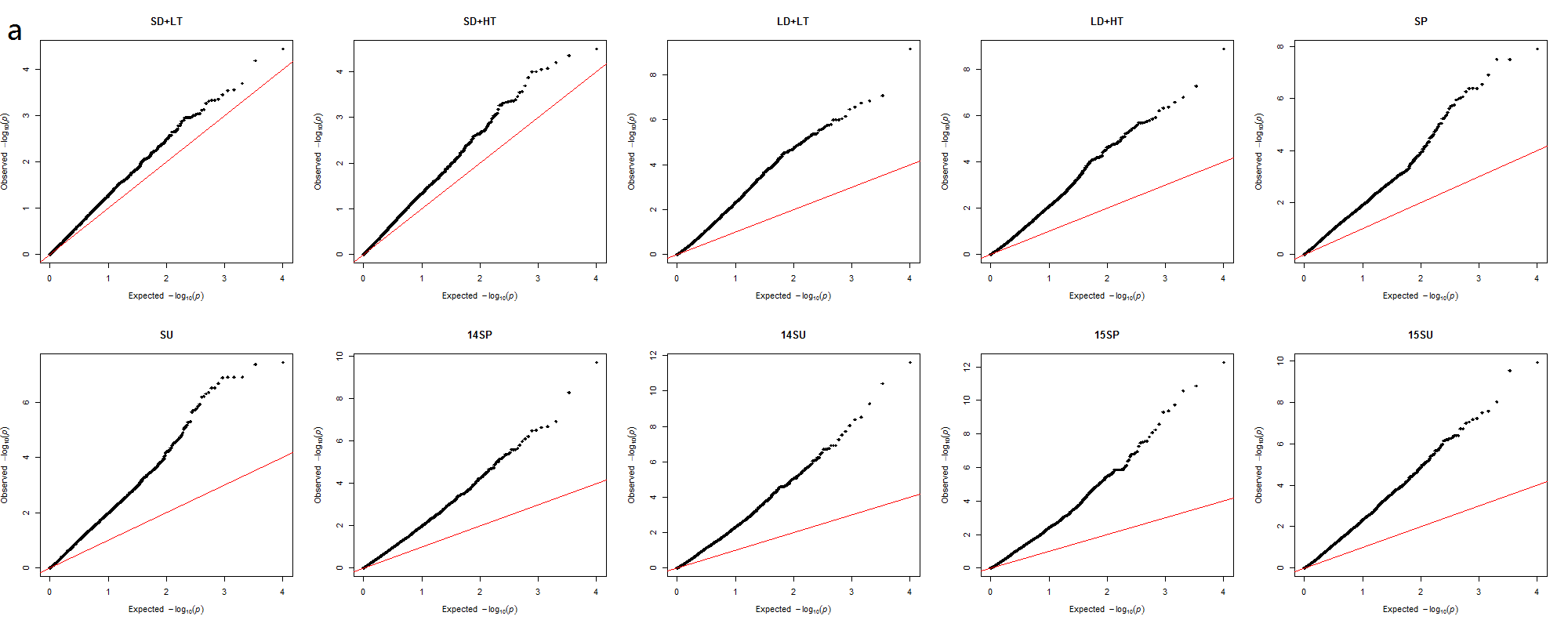


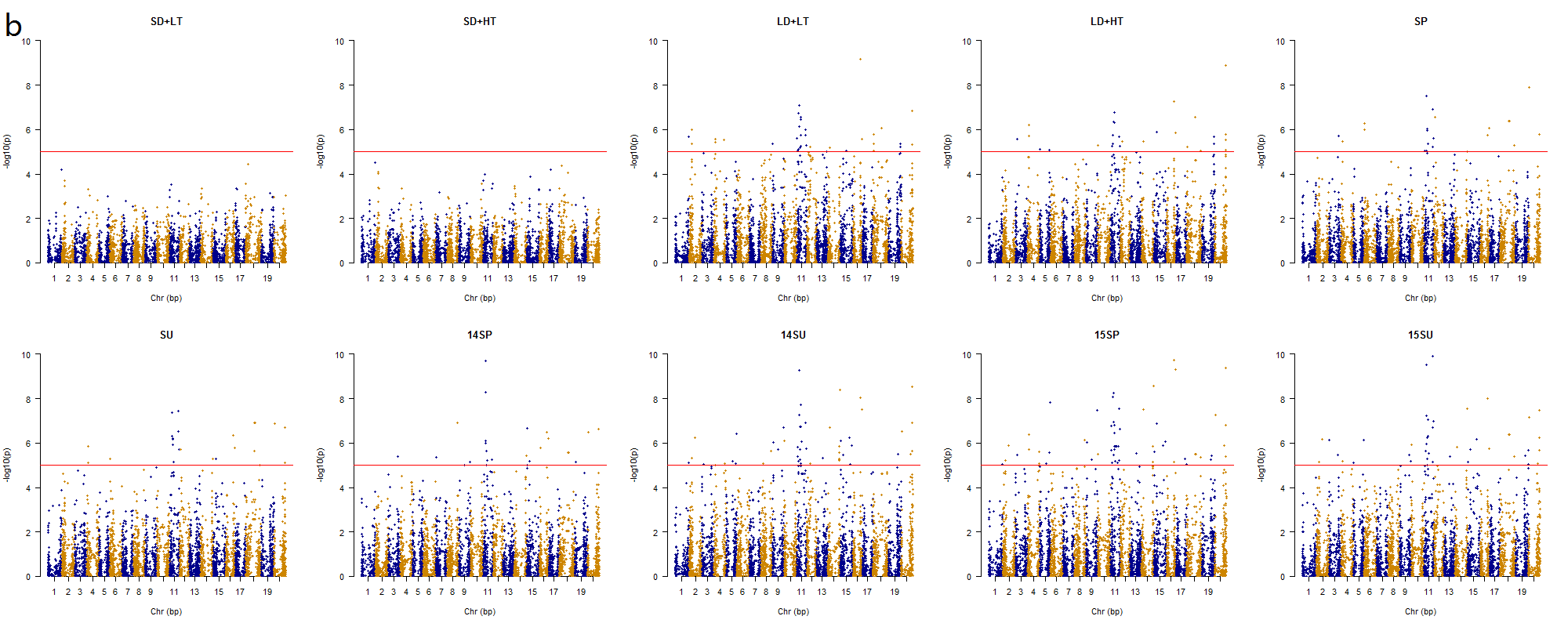


**Figure S3Genome-wide association scan for flowering time in different environments using SNPs. (a) The Quantile-Quantile Plot; (b) Manhattan plot for days to flowering.** P-values (negative log-transformed) are shown in the plot relative to their position on each of the 20 chromosomes. The horizontal pink line indicates the genome-wide significant threshold (9.79×10^-6^).
