# Supplementary material for: Association mapping of loci controlling genetic and environmental interaction of soybean flowering time under various photo-thermal conditions
Source: BMC Genomics. 2017 May 26;18:415. doi: 10.1186/s12864-017-3778-3 (PMC5446728; doi:10.1186/s12864-017-3778-3)
Supplement: Supplementary file 6 — Manhattan plot for days to flowering in the association panel in different environments using SSRs. (a) Quantile-Quantile Plot (b) Manhattan plot for days to flowering. P-values (negative log-transformed) are shown in the plot relative to their genetic positions, the horizontal pink line indicates the genome-wide significant threshold (2.86 × 10^−4^). (DOCX 384 kb) [file 12864_2017_3778_MOESM6_ESM.docx]

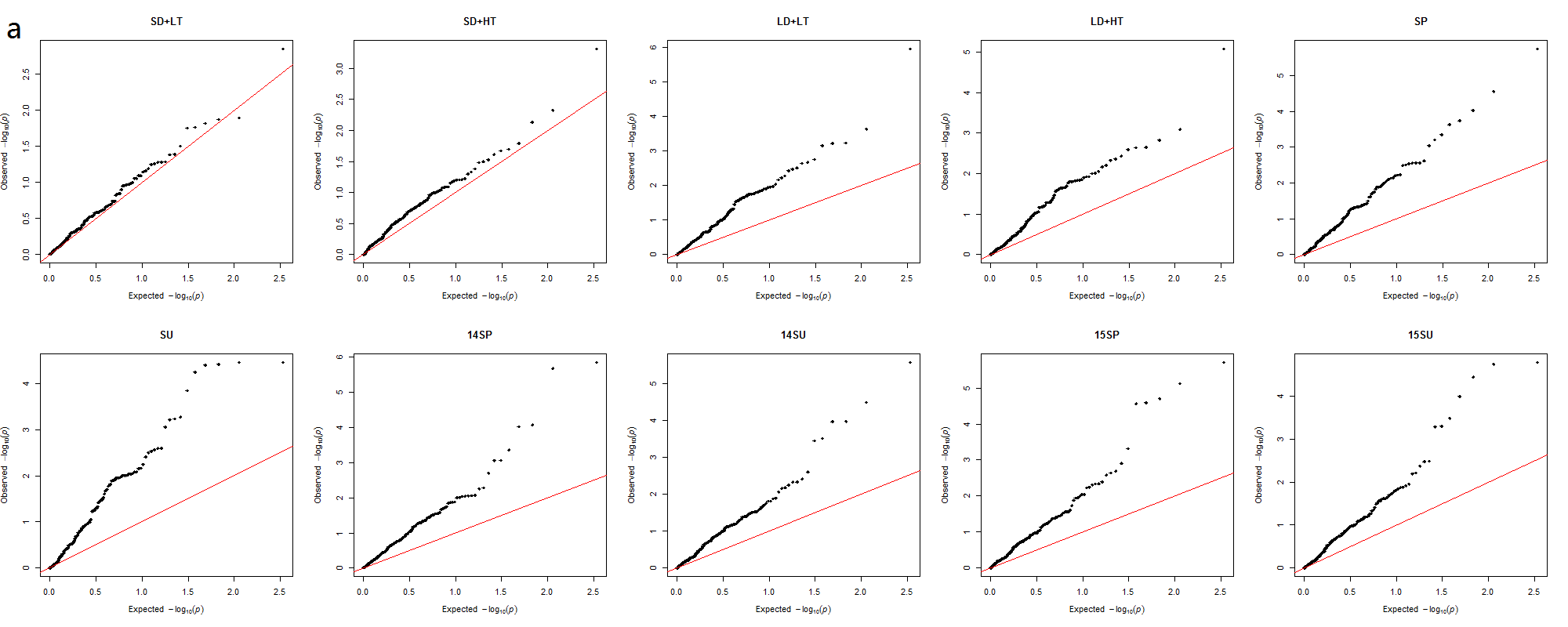


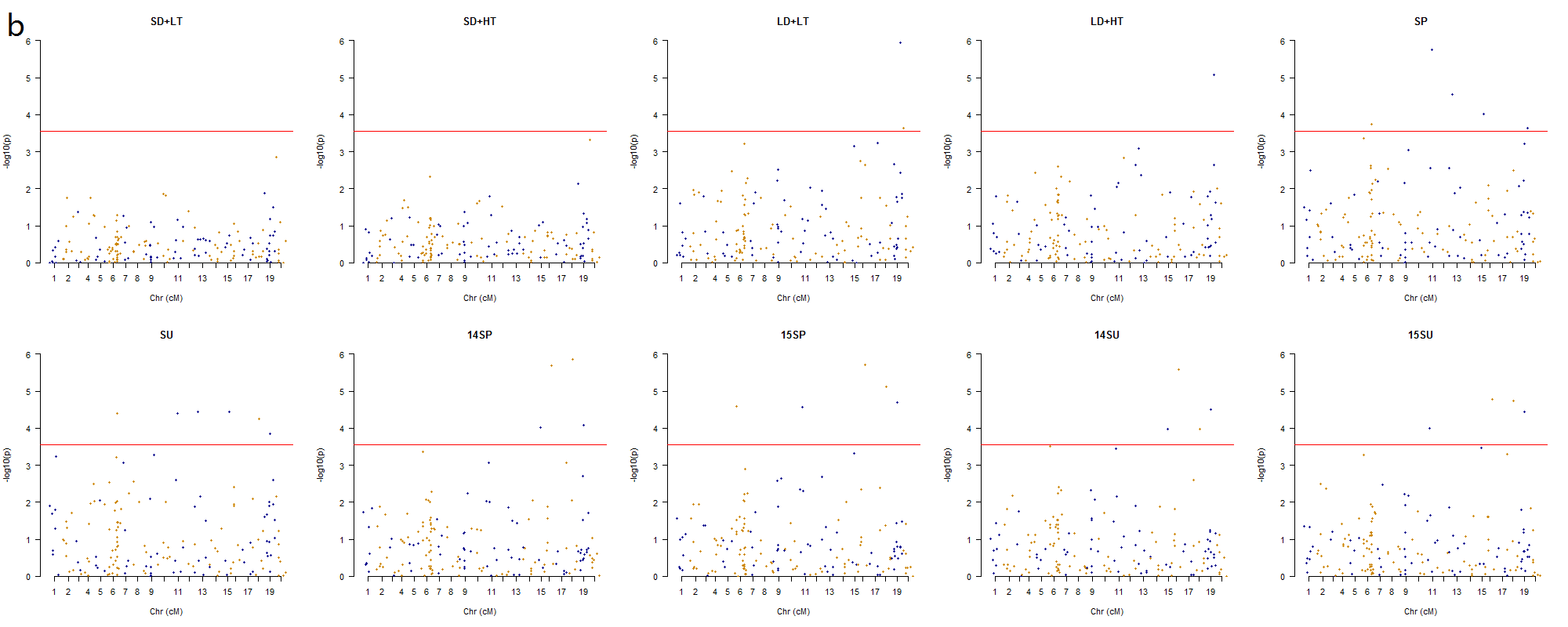


**Figure S4Manhattan plot for days to flowering in the association panel in different environments using SSRs. (a) Quantile-Quantile Plot (b)Manhattan plot for days to flowering.** P-values (negative log-transformed) are shown in the plot relative to their genetic position, the horizontal pink line indicates the genome-wide significant threshold (2.86×10^-4^).
