# Supplementary material for: Association mapping of loci controlling genetic and environmental interaction of soybean flowering time under various photo-thermal conditions
Source: BMC Genomics. 2017 May 26;18:415. doi: 10.1186/s12864-017-3778-3 (PMC5446728; doi:10.1186/s12864-017-3778-3)
Supplement: Supplementary file 7 — The mean flowering time of the accession carrying different alleles. (DOCX 21 kb) [file 12864_2017_3778_MOESM7_ESM.docx]

**Table S3 The mean flowering time of the cultivars carrying different alleles**

| Locus | Allele | Freqency | LD+LT(d) | LD+HT(d) | SP(d) | SU(d) | 14SP(d) | 14Su(d) | 15SP(d) | 15Su(d) |
| --- | --- | --- | --- | --- | --- | --- | --- | --- | --- | --- |
|  |  |  |  |  |  |  |  |  |  |  |
| Gm11_ | CC (Minor) | 38 | 131.7 | 84.1 | 71 | 47.2 | 78.2 | 53.5 | 75.5 | 49.5 |
| 10847172 | TT (Major) | 53 | 93.2 | 64.3 | 49.2 | 36.6 | 45.8 | 36.3 | 42.1 | 33.6 |
|  | Δm | | 38.5 | 19.8 | 21.8 | 10.6 | 32.4 | 17.2 | 33.4 | 15.9 |
| Gm11_ | CC (Minor) | 34 | 157.2 | - | 94.2 | 57.5 | 104.1 | 67 | 98 | 60 |
| 33034954 | TT (Major) | 57 | 93.5 | - | 48.2 | 36.4 | 44.9 | 35.7 | 41.4 | 33.3 |
|  | Δm | | 63.7 | - | 46 | 21.1 | 59.2 | 31.3 | 56.6 | 26.7 |
| Gm16_ | CC (Minor) | 34 | 135.5 | 89.7 | 74.6 | 48.4 | 80.5 | 53.8 | 74.7 | 49.3 |
| 30766209 | TC (Major) | 57 | 78 | 54.5 | 39.5 | 32.6 | 34.3 | 30.4 | 31.5 | 29 |
|  | Δm | | 57.5 | 35.2 | 35.1 | 15.8 | 46.2 | 23.4 | 43.2 | 20.3 |
| Gm20_ | GG (Minor) | 8 | 128.5 | 87.1 | 71 | 47.1 | 73.9 | 51 | 69.4 | 46.3 |
| 43146832 | AA (Major) | 83 | 79.8 | 54.3 | 40 | 32.6 | 34.5 | 30.7 | 32 | 29.5 |
|  | Δm | | 48.7 | 32.8 | 31 | 14.5 | 39.4 | 20.3 | 37.4 | 16.8 |
| Gm20_ | CC (Minor) | 38 | - | - | 99 | 58 | 98.8 | 64.2 | 94.1 | 58.7 |
| 3880320 | TT (Major) | 53 | - | - | 49 | 37 | 46.2 | 36.7 | 43.1 | 34.1 |
|  | Δm | | - | - | 50 | 21 | 52.6 | 27.5 | 51 | 24.6 |

LD, 16 h of light; LT, low temperature (spring sowing); HT, high temperature (summer sowing); SP, Spring sowing season with natural day-length in 2010 pot experiment; SU, Summer sowing season with natural day-length in 2010 pot experiment; 14SP, Spring sowing in 2014 field experiment; 14SU, Summer sowing season in 2014 field experiment; 15SP, Spring sowing season in 2015 field experiment; 15SU, Summer sowing season in 2015 field experiment; Δm , The difference of means between genotypes and phenotypes.

- represents the effect of loci is not significant under the respective photo-thermal condition.
