# Supplementary material for: Association mapping of loci controlling genetic and environmental interaction of soybean flowering time under various photo-thermal conditions
Source: BMC Genomics. 2017 May 26;18:415. doi: 10.1186/s12864-017-3778-3 (PMC5446728; doi:10.1186/s12864-017-3778-3)
Supplement: Supplementary file 8 — The plot of the interactions between significant loci with the flowering time and environment detected by the QTXNetwork. Red columns represent general QTX effects for all six environments. The green lines denote the n-th environment-specific effect. 1, SD + LT condition; 2, SP condition; 3, LD + LT condition; 4, SD + HT condition; 4–42, Gm04_4497001; 4–154, Gm04_42153936; 11–190, Gm11_36124908; 15–116, Gm15_11855585; 16–152, Gm16_30766209; 19–208, Gm19_44042544; 19–243, Gm19_47514601. (DOCX 407 kb) [file 12864_2017_3778_MOESM8_ESM.docx]

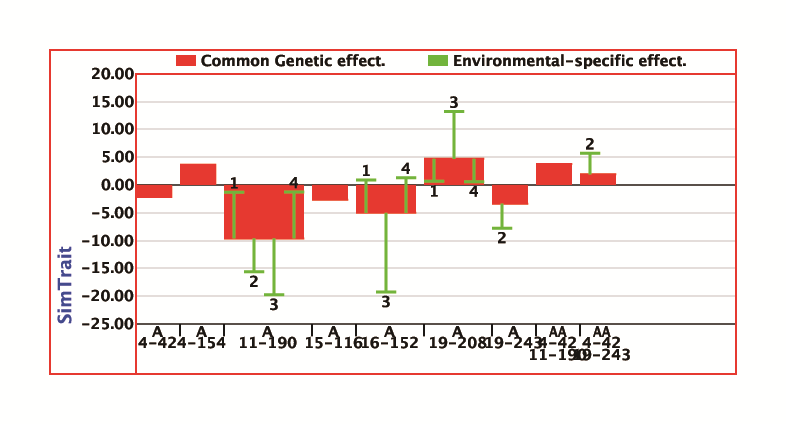


**Figure S5 The plot of the interactions between significant loci with the flowering time and environment detected by the QTXNetwork.** Red columns represent general QTX effects for all six environments. The green lines denote the n-th environment-specific effect. 1, SD + LT condition; 2, SP condition; 3, LD + LT condition; 4, SD + HT condition; 4-42, Gm04_4497001; 4-154, Gm04_42153936; 11-190, Gm11_36124908; 15-116, Gm15_11855585; 16-152, Gm16_30766209; 19-208, Gm19_44042544; 19-243, Gm19_47514601.
