# Supplementary material for: Association mapping of loci controlling genetic and environmental interaction of soybean flowering time under various photo-thermal conditions
Source: BMC Genomics. 2017 May 26;18:415. doi: 10.1186/s12864-017-3778-3 (PMC5446728; doi:10.1186/s12864-017-3778-3)
Supplement: Supplementary file 9 — The significant loci associated with flowering time and related candidate genes. (DOCX 27 kb) [file 12864_2017_3778_MOESM9_ESM.docx]

**Table S4 The significant loci associated with flowering time and related candidate genes**

| Marker | Chr | Position | Candidate gene | Annotation |
| --- | --- | --- | --- | --- |
| Gm01_53278791 | Gm01 | 53278791 | Glyma01g41590; Glyma01g41850;  Glyma01g42230 | ubiquitin-protein ligases(HOS1); DNAJ heat shock N-terminal domain-containing protein;  AT-hook motif nuclear-localized protein 22 |
| Gm01_53675540 | Gm01 | 53675540 | Glyma01g42110 | SRF-type transcription factor (DNA-binding and dimerisation domain) |
|  |  |  |  | MADS BOX PROTEIN |
| Gm02_10536842 | Gm02 | 10536842 | Glyma02g12220 | NAC-like, activated by AP3/PI |
| Gm02_11998056 | Gm02 | 11998056 | Glyma02g13401; Glyma02g13420; | K-box region and MADS-box transcription factor family protein( |
|  |  |  |  | AGL2,SEP1); K-box region and MADS-box; |
| Gm02_22829006 | Gm02 | 22829006 |  |  |
| Gm03_1077329 | Gm03 | 1077329 | Glyma03g01540 | Myb domain protein 17; |
| Gm03_5502496 | Gm03 | 5502496 | Glyma03g05180 | SAM dependent carboxyl methyltransferase" |
| Gm03_36634361 | Gm03 | 36634361 | Glyma03g28740 | Gibberellin-regulated family protein |
| Gm03_38526701 | Gm03 | 38526701 | Glyma03g30340; Glyma03g30460;  Glyma03g30910 | zinc finger protein 8;alpha/beta-Hydrolases superfamily protein;  Plant protein of unknown function (DUF828) |
| Gm04_4497001 | Gm04 | 4497001 | Glyma04g06240 | CONSTANS-like 4 |
| Gm04_38840391 | Gm04 | 38840391 | Glyma04g33270; Glyma04g33410; | No Apical Meristem (NAM) protein; cycling DOF factor 3; |
|  |  |  | Glyma04g33110 | CCT motif -containing response regulator protein(TOC1) |
| Gm04_42951376 | Gm04 | 42951376 | Glyma04g36450; Glyma04g36620;  Glyma04g36630 | Leucine-rich repeat protein kinase family protein(EVR,SOBIR1);  relative of early flowering 6(REF6);relative of early flowering 6(REF6) |
| Gm04_46390533 | Gm04 | 46390533 | Glyma04g40090; Glyma04g40150;  Glyma04g40640 | double-stranded DNA binding;methyltransferase 1;pseudo-response regulator 5 |
| Gm05_682648 | Gm05 | 682648 | Glyma05g00880 | CCT motif -containing response regulator protein (TOC1) |
| Gm05_1705841 | Gm05 | 1705841 | Glyma05g02220 | Dof-type zinc finger DNA-binding family protein |
| Gm05_26685967 | Gm05 | 26685967 | Glyma05g21880; Glyma05g21790 | Tetratricopeptide repeat (TPR)-like superfamily protein;FRIGIDA-like protein |
| Gm05_38636402 | Gm05 | 38636402 | Glyma05g34280; Glyma05g34030 | Zinc finger, C2H2 type(SUF4); |
|  |  |  |  | PEBP (phosphatidylethanolamine-binding protein) family protein(MFT) |
| Gm05_38636402 | Gm05 | 38636402 |  |  |
| Gm05_40349605 | Gm05 | 40349605 | Glyma05g36400; Glyma05g36560 | non-intrinsic ABC protein 7;Transducin/WD40 repeat-like superfamily protein |
| Gm06_2086304 | Gm06 | 2086304 | Glyma06g02990 | K-box region MADS BOX PROTEIN(AP3) |
| Gm06_2253042 | Gm06 | 2253042 | Glyma06g02990 | K-box region MADS BOX PROTEIN(AP3) |
| Satt422 | Gm06 | 7227638 |  |  |
| Gm07_3143196 | Gm07 | 3143196 | Glyma07g04290 | growth-regulating factor 5 |
| Gm08_11052135 | Gm08 | 11052135 | Glyma08g15610 | Amino acid kinase family protein |
| Gm08_40882335 | Gm08 | 40882335 | Glyma08g40860 | ubiquiting-conjugating enzyme 2 |
| Gm09_24238724 | Gm09 | 24238724 |  |  |
| Gm09_39822766 | Gm09 | 39822766 | Glyma09g33340; Glyma09g33350 | Di-glucose binding protein with Kinesin motor domain;  Dof-type zinc finger DNA-binding family protein |
| Gm09_43508261 | Gm09 | 43508261 | Glyma09g37810; Glyma09g38370 | Protein kinase superfamily protein;Integrase-type DNA-binding superfamily protein |
| Gm10_2317882 | Gm10 | 2317882 | Glyma10g02840 | Receptor-like kinase in in flowers 3(RKF3) |
| Gm11_1161553 | Gm11 | 1161553 | Glyma11g01640; Glyma11g01700; | AP2 domain; Myb-like DNA-binding domain; AP2 domain |
|  |  |  | Glyma11g02060; Glyma11g02140 |  |
| Gm11_3950213 | Gm11 | 3950213 | Glyma11g05550; Glyma11g05720 | Myb-like DNA-binding domain; AP2 domain |
| Gm11_4519147 | Gm11 | 4519147 | Glyma11g06480; Glyma11g06300 | Tetratricopeptide repeat (TPR)-like superfamily protein;  Dof-type zinc finger DNA-binding family protein |
| Gm11_5065170 | Gm11 | 5065170 | Glyma11g06840; Glyma11g12460 | Sugar transporter; Myb-like DNA-binding domain |
| Gm11_6512939 | Gm11 | 6512939 | Glyma11g09280; Glyma11g09400 | RING/U-box superfamily protein;FAR1-related sequence 5 |
| Satt197 | Gm11 | 8879480 | Glyma11g12460 | Myb-like DNA-binding domain |
| Gm11_10847172 | Gm11 | 10847172 | Glyma11g15504 | CCT motif family protein |
| Gm11_11572077 | Gm11 | 11572077 | Glyma11g14920; Glyma11g15650; | Dof domain, Zinc finger; AP2 domain ; Dof domain |
|  |  |  | Glyma11g15760; Glyma11g16050 | Zinc finger; bZIP transcription factor |
| Gm11_16492046 | Gm11 | 16492046 | Glyma11g19830 | C2H2-type zinc finger family protein |
| Gm11_17237725 | Gm11 | 17237725 | Glyma11g20220 | ABC transporter family protein |
| Gm11_21023332 | Gm11 | 21023332 | Glyma11g20420; Glyma11g20240 | 3\'-5\'-exoribonuclease family protein;POX (plant homeobox) family protein |
| Gm11_33034954 | Gm11 | 33034954 | Glyma11g31940 | Auxin response factor 8 |
| Gm11_33555216 | Gm11 | 33555216 | Glyma11g31940 | Auxin response factor 8 |
| Gm11_36174968 | Gm11 | 36174968 | Glyma11g34250 | AT-hook protein of GA feedback 2; |
| Gm12_5786241 | Gm12 | 5786241 | Glyma12g07926; Glyma12g08290 | CCT motif family protein; ABC transporter family protein |
| Gm12_13354287 | Gm12 | 13354287 |  |  |
| Satt586 | Gm13 | 11639980 | Glyma13g09980 | MYB domain protein 5 |
| Gm13_23509779 | Gm13 | 23509779 | Glyma13g19910 | Homeodomain-like superfamily protein |
| Gm13_39307253 | Gm13 | 39307253 | Glyma13g38140; Glyma13g3825 | Basic helix-loop-helix (bHLH) DNA-binding superfamily |
|  |  |  |  | protein; B-box type zinc finger protein with CCT domain |
|  |  |  |  | (CO) |
| Gm14_7302299 | Gm14 | 7302299 | Glyma14g09231; Glyma14g09340 | Cold regulated gene 27; C2H2-like zinc finger protein |
| Gm14_7302299 | Gm14 | 7302299 |  |  |
| Gm14_44697544 | Gm14 | 44697544 |  |  |
| Gm14_45457682 | Gm14 | 45457682 | Glyma14g36100; Glyma14g36150 | vernalization5/VIN3-like;GATA-type zinc finger protein with TIFY domain |
| Gm14_49107190 | Gm14 | 49107190 | Glyma14g40450 | C2H2-like zinc finger protein |
| Gm15_1265753 | Gm15 | 1265753 | Glyma15g02030; Glyma15g02040 | Tetratricopeptide repeat (TPR)-like superfamily protein;  phytochrome-associated protein 2 |
| Gm15_35867161 | Gm15 | 35867161 |  |  |
| Gm15_45004801 | Gm15 | 45004801 |  |  |
| Gm16_5773005 | Gm16 | 5773005 |  |  |
| SSRFT | Gm16 | 30741600 | Glyma16g26660; Glyma16g26690 | PEBP(phosphatidylethanolamine-binding protein) family protein; |
|  |  |  |  | PEBP (phosphatidylethanolamine-binding protein) family protein |
| Gm16_35700223 | Gm16 | 35700223 | Glyma16g32540; Glyma16g32550; | AGAMOUS-like 6; gibberellin 20 oxidase 2; basic |
|  |  |  | Glyma16g32610 | Helix-loop-helix (bHLH) DNA-binding superfamily protein |
| Gm17_37574384 | Gm17 | 37574384 | Glyma17g33480 | G-protein-coupled receptor 1 |
| Gm17_41063513 | Gm17 | 41063513 | Glyma17g37580 | Transcriptional factor B3 family protein / auxin-responsive factor AUX/IAA-related |
| Gm18_4324818 | Gm18 | 4324818 | Glyma18g05916; Glyma18g05963 | AGAMOUS-like 80 |
| Gm18_35693915 | Gm18 | 35693915 | Glyma18g3090 | Auxin-responsive family protein |
| Satt564 | Gm18 | 47617795 | Glyma18g39740; Glyma18g3977 | MYB domain protein; MYB-like HTH transcriptional |
|  |  |  |  | regulator family protein |
| Gm19_5195925 | Gm19 | 5195925 | Glyma19g05170 | B-box type zinc finger protein with CCT domain(CO) |
| Gm19_35449676 | Gm19 | 35449676 | Glyma19g28250 | Transducin/WD40 repeat-like superfamily protein |
| Gm19_39723056 | Gm19 | 39723056 | Glyma19g31720; Glyma19g31960 | INO80 ortholog; Integrase-type DNA-binding superfamily protein |
| sat_113 | Gm19 | 42110332 | Glyma19g34740; Glyma19g34550 | MYB domain protein 15; C2H2 and C2HC zinc fingers |
|  |  |  |  | superfamily protein |
| Satt664 | Gm19 | 46109700 | Glyma19g39460 | B-box type zinc finger protein with CCT domain(CO) |
| Gm19_46761039 | Gm19 | 46761039 | Glyma19g40090; Glyma19g40630 | Ethylene response sensor 1 phytochrome interacting factor 3 |
| Satt229 | Gm19 | 47049074 | Glyma19g40630; Glyma19g40640; | MYB-like transcription factor family protein; gibberellin |
|  |  |  | Glyma19g40980 | 2-oxidase 6; phytochrome interacting factor 3 |
| Gm19_47514601 | Gm19 | 47514601 | Glyma19g40970; Glyma19g41210; | Phytochromeinteracting factor 3; PhytochromeA ; |
|  |  |  | Glyma19g4126 | Phytochromeinteracting factor 3 |
| Gm19_49786000 | Gm19 | 49786000 | Glyma19g44310; Glyma19g44670 | Auxin-responsive GH3 family protein；Dof-type zinc finger DNA-binding family protein |
| Satt571 | Gm20 | 1291809 | Glyma20g01610; Glyma20g01760 | MYB domain protein 3; Auxin efflux carrier family protein |
| Gm20_3880320 | Gm20 | 3880320 | Glyma20g03988 | phytochrome and flowering time regulatory protein (PFT1) |
| Gm20_37857633 | Gm20 | 37857633 | Glyma20g28670; Glyma20g29210; Glyma20g29250 | TCP family transcription factor;gibberellin 20 oxidase 2;  K-box region and MADS-box transcription factor family protein |
| Gm20_43146832 | Gm20 | 43146832 | Glyma20g35020; Glyma20g35220 | COP1-interacting protein-related; cryptochrome 2 |
| Gm20_44260228 | Gm20 | 44260228 | Glyma20g35910 | Dof-type zinc finger DNA-binding family protein |
